# Meta-Analysis of Altered Gut Microbiota Reveals Microbial and Metabolic Biomarkers for Colorectal Cancer

**DOI:** 10.1128/spectrum.00013-22

**Published:** 2022-06-29

**Authors:** Nagavardhini Avuthu, Chittibabu Guda

**Affiliations:** a Department of Genetics, Cell Biology and Anatomy, University of Nebraska Medical Center, Omaha, Nebraska, USA; b Center for BioInformatics Research and Innovation (CBIRI), University of Nebraska Medical Center, Omaha, Nebraska, USA; Lerner Research Institute

**Keywords:** biomarkers, colorectal cancer, gut dysbiosis, meta-analysis, microbial metabolites, microbiome

## Abstract

Colorectal cancer (CRC) is the second leading cause of cancer mortality worldwide. The dysbiotic gut microbiota and its metabolite secretions play a significant role in CRC development and progression. In this study, we identified microbial and metabolic biomarkers applicable to CRC using a meta-analysis of metagenomic datasets from diverse geographical regions. We used LEfSe, random forest (RF), and co-occurrence network methods to identify microbial biomarkers. Geographic dataset-specific markers were identified and evaluated using area under the ROC curve (AUC) scores and random effect size. Co-occurrence networks analysis showed a reduction in the overall microbial associations and the presence of oral pathogenic microbial clusters in CRC networks. Analysis of predicted metabolites from CRC datasets showed the enrichment of amino acids, cadaverine, and creatine in CRC, which were positively correlated with CRC-associated microbes (Peptostreptococcus stomatis, Gemella morbillorum, Bacteroides fragilis, *Parvimonas* spp., Fusobacterium nucleatum, Solobacterium moorei, and Clostridium symbiosum), and negatively correlated with control-associated microbes. Conversely, butyrate, nicotinamide, choline, tryptophan, and 2-hydroxybutanoic acid showed positive correlations with control-associated microbes (*P* < 0.05). Overall, our study identified a set of global CRC biomarkers that are reproducible across geographic regions. We also reported significant differential metabolites and microbe-metabolite interactions associated with CRC. This study provided significant insights for further investigations leading to the development of noninvasive CRC diagnostic tools and therapeutic interventions.

**IMPORTANCE** Several studies showed associations between gut dysbiosis and CRC. Yet, the results are not conclusive due to cohort-specific associations that are influenced by genomic, dietary, and environmental stimuli and associated reproducibility issues with various analysis approaches. Emerging evidence suggests the role of microbial metabolites in modulating host inflammation and DNA damage in CRC. However, the experimental validations have been hindered by cost, resources, and cumbersome technical expertise required for metabolomic investigations. In this study, we performed a meta-analysis of CRC microbiota data from diverse geographical regions using multiple methods to achieve reproducible results. We used a computational approach to predict the metabolomic profiles using existing CRC metagenomic datasets. We identified a reliable set of CRC-specific biomarkers from this analysis, including microbial and metabolite markers. In addition, we revealed significant microbe-metabolite associations through correlation analysis and microbial gene families associated with dysregulated metabolic pathways in CRC, which are essential in understanding the vastly sporadic nature of CRC development and progression.

## INTRODUCTION

CRC is the third most diagnosed and the second leading cause of cancer-related deaths for men and women combined, globally (1). Genetic and environmental factors influence CRC incidences. Most CRCs are sporadic (70%), while about 25% are familial, and about 5% are hereditary (2). Environmental factors consistently associated with sporadic CRC incidences include a low-fiber diet, tobacco, alcohol, lack of physical activity, obesity, age, and diabetes mellitus (3–8) which could modify gut microbial composition and function (9–11). Gut microbiota is a complex and dynamic microbial community with diverse functions that significantly contribute to human health and metabolic and immune functions. Dysbiosis of gut microbiota is associated with numerous gastrointestinal and extraintestinal disorders, including cancers (12–14). It could contribute to the etiology of CRC by altering the inflammatory, genomic, and metabolic processes in the host. To date, only a few pathogenic species such as F. nucleatum, B. fragilis, and Escherichia coli were experimentally shown to be involved in CRC carcinogenesis through inflammatory and genotoxic activities (15–17). The dysbiotic gut microbiota also promotes inflammation and alters the host metabolism through its metabolite secretions (18). For instance, the red meat diet enriches the sulfate-reducing gut bacteria that are involved in the production of hydrogen sulfide, a genotoxic agent (19, 20). Long-term dependence on sulfur microbial diet is associated with increased CRC risk (21).

Several studies have explored the gut microbial composition to identify CRC biomarkers and linked pathogenic bacteria such as B. fragilis, F. nucleatum, Streptococcus bovis, E. coli, Enterococcus faecalis, and *Porphyromonas* spp. to CRC (7, 22–24). However, consistent CRC biomarkers are lacking as most of the studies are associated with specific cohorts that are highly influenced by dietary factors. For example, increased CRC risk in African Americans was shown to be associated with secondary bile acids production by enriched *Bacteroides* under a high-fat and low-fiber diet (25). Conversely, decreased CRC risk in native Africans was associated with increased short-chain fatty acid (SCFAs) production by enriched *Prevotella* members under high-fiber diet conditions (25). The altered gut microbial metabolite profiles, such as a decrease in SCFAs (acetate, propionate, and butyrate) and an increase in secondary bile acids are shown to promote carcinogenesis through a proinflammatory mechanism (26), and diet-derived microbial metabolites, such as N-nitroso compounds, azo compounds, and nitrates lead to genotoxic and carcinogenic effects in the host (27). Despite the vital role played by the microbe-derived metabolites in CRC, global biomarkers are not available due to cumbersome experimental limitations that involve testing in animal models.

We identified a set of global biomarkers for CRC in this study through a comprehensive meta-analysis of existing CRC datasets from diverse geographical regions and identified a novel CRC biomarker, A. onderdonkii. Using a computational approach, we predicted the metabolomic profiles of CRC datasets and identified metabolite biomarkers, which are consistent with previous metabolomic studies, such as the enrichment of butanoic acid in controls but different amino acids in the CRC cohort. And in this study, we also showed the significant microbial and metabolite correlations in CRC pathogenesis. Next, we identified potential functional capabilities in CRC pathogens and their differences across the geographical regions based on gene family analysis. This study enhances our understanding of the role of CRC-associated microbes and their metabolites in CRC development and progression.

## RESULTS

### Composition and diversity of gut microbial communities associated with CRC.

**FIG 1:**
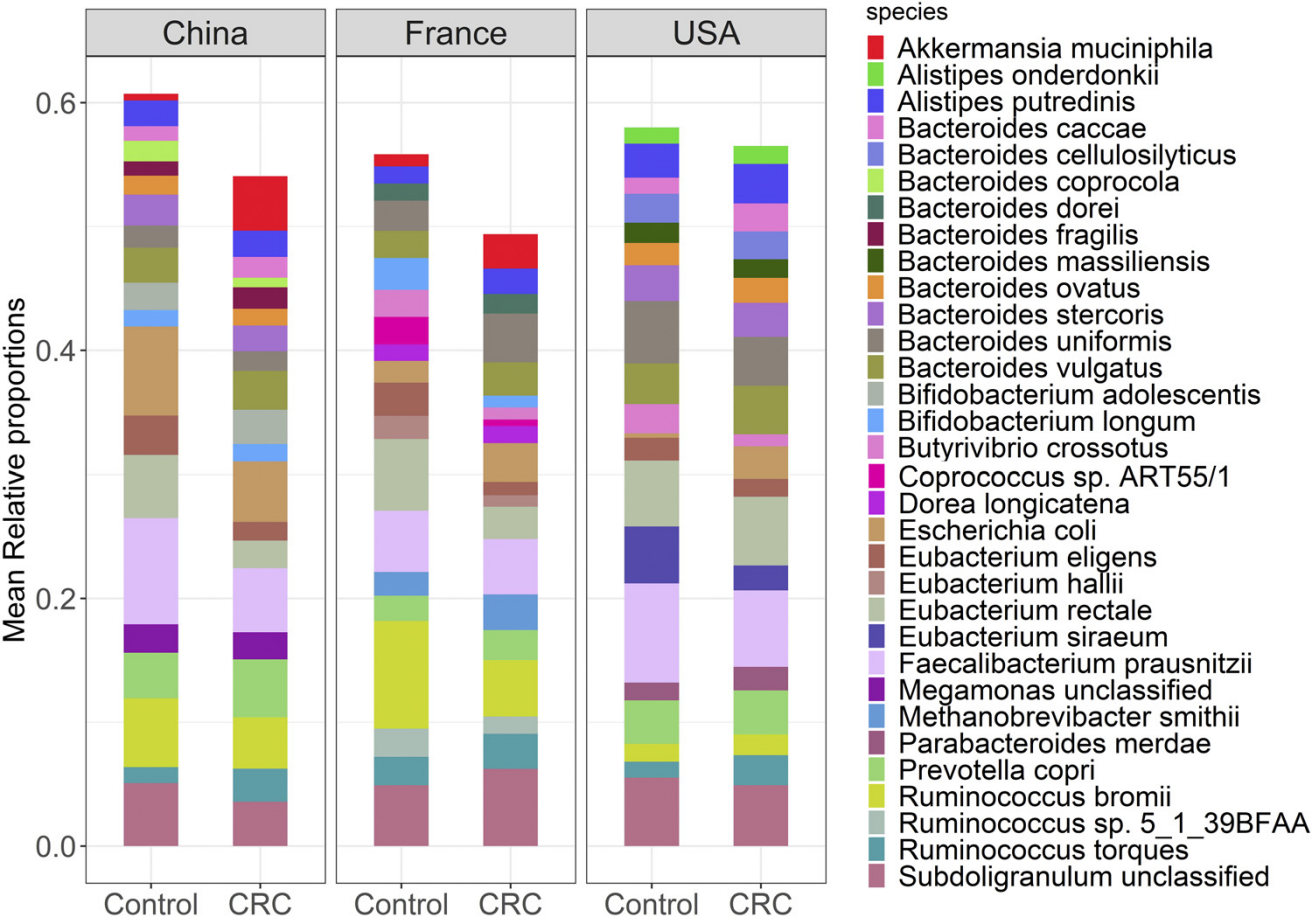
Stacked bar plot shows the mean relative proportions of the top 20 species-level taxa of gut microbial communities in CRC and healthy controls.

The taxonomic composition of CRC gut communities was analyzed using publicly available shotgun metagenomic sequencing data from three different geographical regions, the USA, China, and France (Table S1). High-quality sequencing reads were selected after preprocessing and quality control and then quantified for taxonomic composition using *MetaPhlAn* software (Data Set S1). We identified archaea, bacteria, eukaryotes, and viruses in most of the samples. However, bacterial taxa dominated with more than 97% of all species identified in each sample. We selected a total of 418, 410, and 469 microbial species with average relative abundance >0.1% from the samples of the USA, China, and France datasets, respectively. Mean relative abundances of the top 20 species covered >50% of total microbial abundance (Fig. 1, Data Set S2). And most of these species, including *Bacteroides* spp., and *Eubacterium* spp., belong to phyla Bacteroidetes and Firmicutes, respectively. High proportions of Faecalibacterium prausnitzii, Eubacterium rectale, and Eubacterium eligens were observed in the control group compared to the CRC group, whereas high proportions of Prevotella copri, Ruminococcus torques, and Bacteroides vulgatus were observed in CRC group compared to control group across the three geographic regions. At the species level, the alpha diversity of metagenomes was not significantly different between CRC and control groups across geographic regions (Fig. S1A). The species diversity in CRC and control groups was independent of potential confounding factors like age, BMI, and sex, as shown in Fig. S1B. The non-metric multidimensional scaling (NMDS) along with PERMANOVA evaluation based on Bray-Curtis dissimilarity measures showed significant differences between CRC and control metagenomes in France and China datasets and no differences between CRC and control metagenomes in the USA dataset (*P* < 0.05) (Fig. S2A to D). Similar comparisons based on the Bray-Curtis dissimilarity measures showed significant differences in CRC and control metagenomes across the geographical regions (*P* < 0.05) (Fig. S3A and B).

### Altered microbial associations in CRC across geographic regions.

Microbial associations in CRC were analyzed at the species level using co-occurrence networks, *LEfSe* algorithm, and RF methods. Differential microbial species were selected based on the geographic region using each method and evaluated using RF models.

In the co-occurrence network analysis, we calculated the correlation coefficients between microbes in CRC cases and controls separately and constructed networks by selecting the significant positive correlations (*r* > 0.4, q < 0.05) for each geographic region. A cluster analysis of networks showed various microbial interactions in CRC and control networks. We identified a cluster formed from oral microbes such as G. morbillorum, Porphyromonas asaccharolytica, *Parivimonas* spp., P. stomatis, Prevotella intermedia, Parvimonas micra, and F. nucleatum in all CRC networks (Fig. S4). The matched cluster was observed in the Chinese control network, however, it showed a high abundance of these microbes in CRC cases compared to healthy controls (Fig. S5), and the results were consistent with previous studies (28, 29).

**FIG 2:**
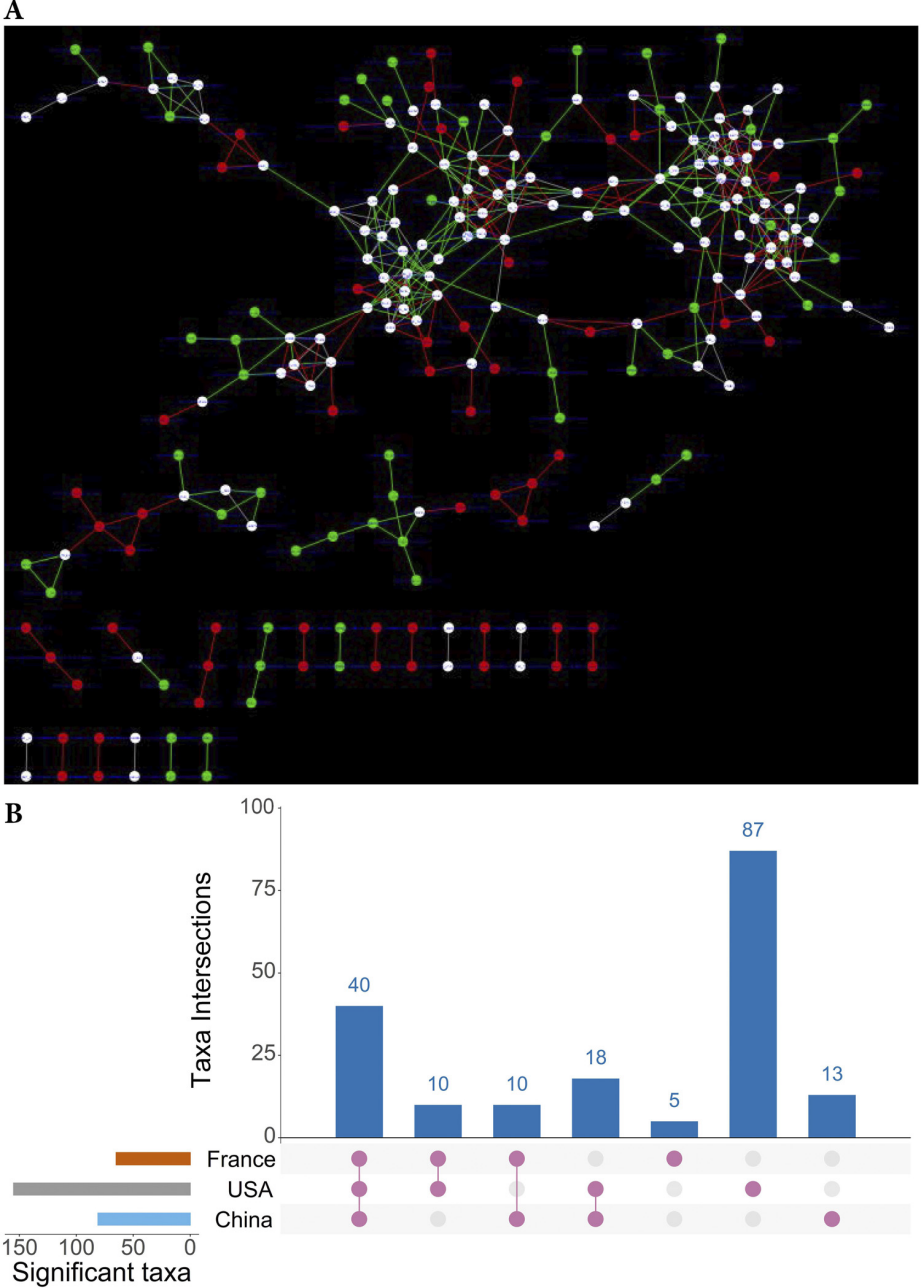
Co-occurrence networks and DyNet dataset-specific markers. (A) DyNet visualization of synchronized CRC and healthy control co-occurrence networks of microbial species from the China dataset. Red nodes and red edges are present only in the CRC network, green nodes and green edges are present only in the control network, and white nodes are present in both. (B) An Upset plot visualization of DyNet dataset-specific markers intersections across France, USA, and China datasets. Each bar represents the number of dataset markers in that category and orange dots below the bar indicates their conservation across the datasets. For instance, the 1st bar shows 40 Dynet dataset-specific markers that are common in all three datasets.

Further, we synchronized the CRC and control networks of each geographic region using *DyNet* software and identified 156, 65, and 81 rewired nodes between CRC and control networks of USA, French, and Chinese datasets, respectively. The rewired node score (D_n_-score) between the two networks indicates the changed interactions among the microbes (e.g., DyNet visualization of synchronized networks for China dataset shown in Fig. 2A, and corresponding unsynchronized networks shown in Fig. S6A and B). Similar networks for the USA and France datasets were shown in Fig. S7 and S8. The rewired nodes were identified based on the D_n_-score (Data Set S3) and compared among the datasets. A set of 40 microbes with changed node perturbations were found in all datasets, and most of these include commensals such as F. pratsnitzii, Roseburia hominis, Eubacteriun halli, and Blautia producta and pathogens, such as S. moorei, Ruminococcus gnavus, and Clostridium *sp*. A high number of unique rewired nodes (87 differential species) were identified from the synchronized network of the USA dataset (Fig. 2B). We selected different sets of rewired nodes for assessing the performance of DyNet markers, and those include differential taxa identified in each geographic region by DyNet (DyNet dataset-specific markers) and a set of 78 rewired nodes that were common in 3 or 2 geographic regions (common DyNet-specific markers).

**FIG 3:**
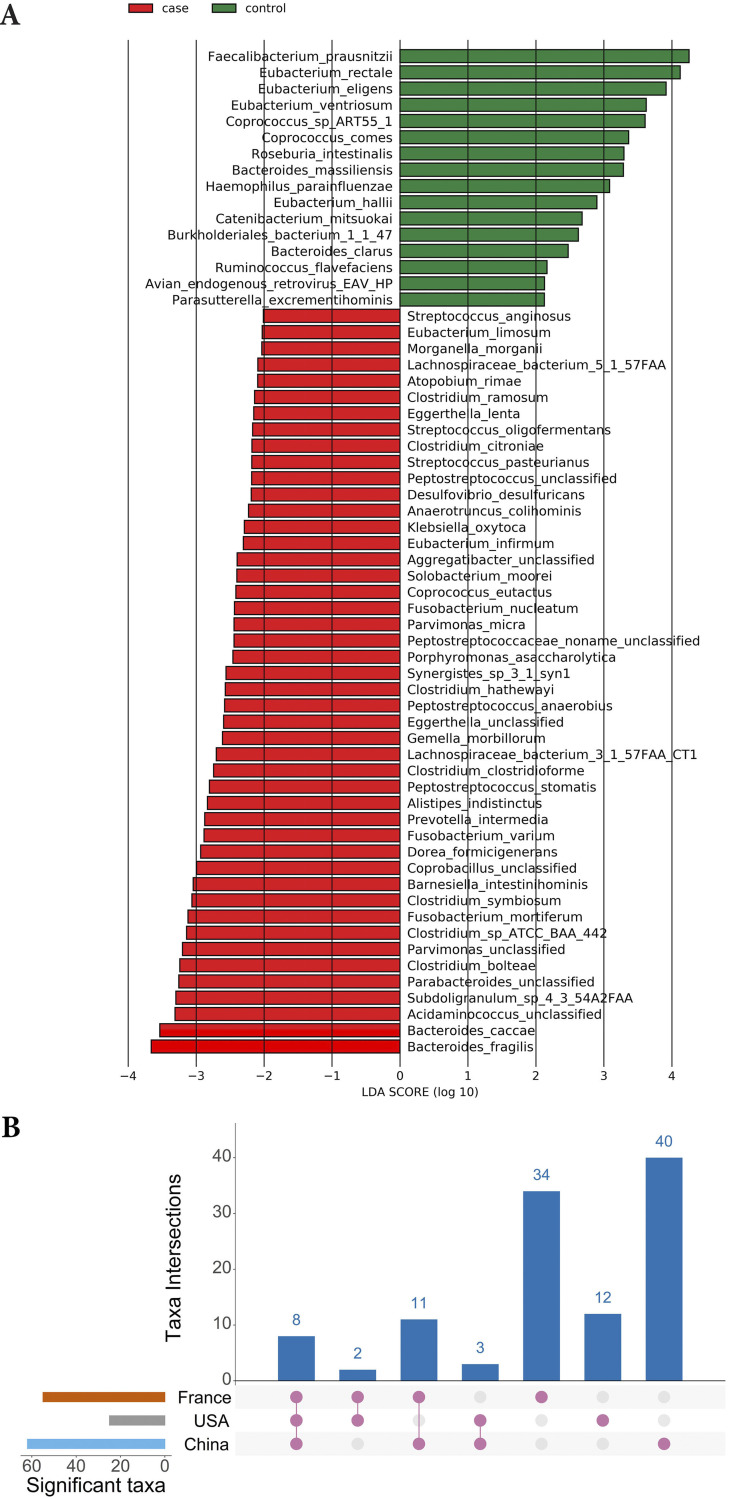
LEfSe analysis. (A) Histograms of differential species in Chinese dataset. CRC enriched species are indicated with a negative LDA score (red), and species enriched in healthy controls are indicated with a positive LDA score (green). Only species with an LDA score >2 at *P* < 0.05 are shown. (B) An Upset plot visualization of LEfSe dataset-specific markers intersections across the three datasets (France, USA, and China). Each bar represents the number of differential species in that category and orange dots below the bar indicate their conservation across the datasets. For instance, the 1st bar shows eight LEfSe identified differential species that are common in all three datasets.

*LEfSe* analysis revealed the significant differences in microbial species between CRC and control groups (LDA score > 2.0, *P* < 0.05) (Fig. 3A and Fig. S9A and B). A total of 110 unique microbial species differed between CRC and control groups among geographic regions. Among those, 8 pathogenic microbes, B. fragilis, C. symbiosum, F. nucleatum, G. morbillorum, *Parvimonas* spp., P. stomatis, P. asaccharolytica, and Prevotella intermedia, were identified as CRC enriched microbes in all three geographic regions. Other species were identified as unique to a geographic region or as common in at least two geographic regions (Fig. 3B, Data Set S3). For performance evaluation, we selected different sets of markers, those included LEfSe identified markers for each geographic region separately (LEfSe dataset-specific markers), and a set of 24 common LEfSe dataset-specific markers present in 3 or 2 geographic regions (common LEfSe-specific markers).

**FIG 4:**
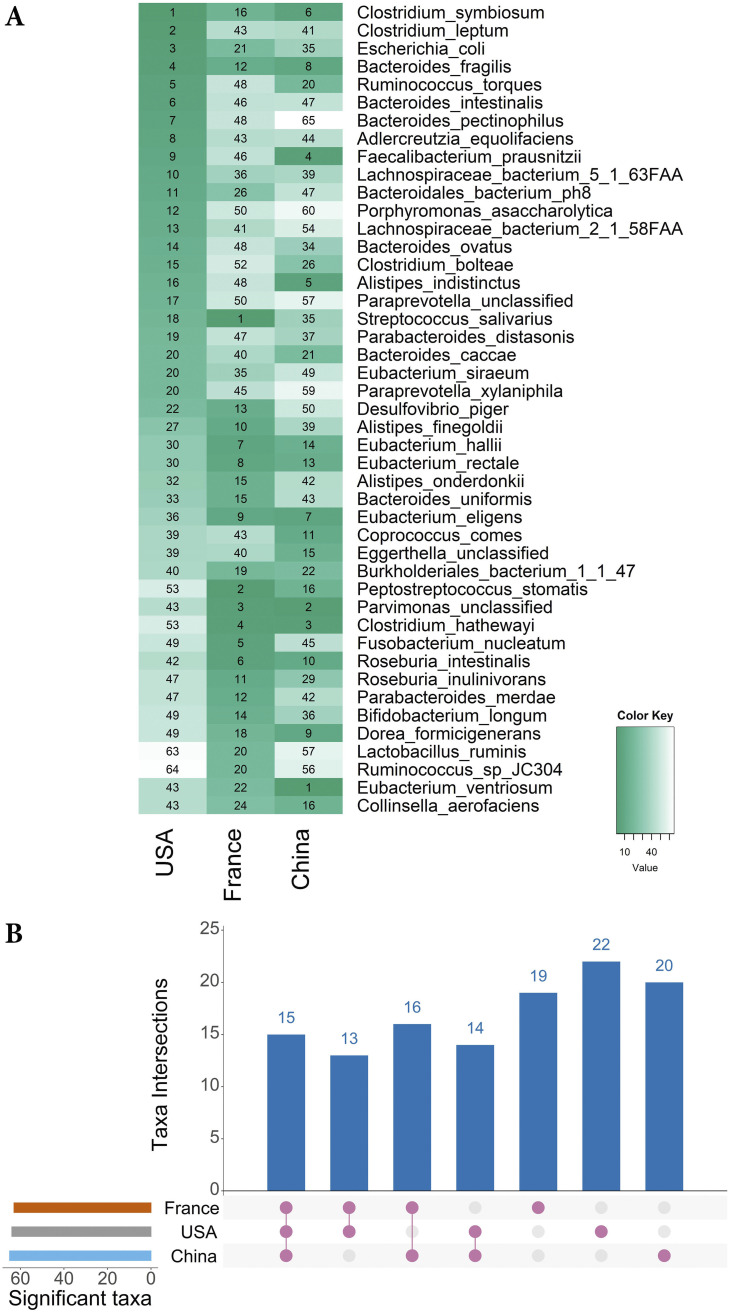
RF identified microbial markers of CRC in USA, French, and Chinese datasets. (A) In the RF cross-validations, the prediction performance of each species was scored based on internal RF rankings. Rankings of the RF-identified species markers with a rank below 20 in at least one dataset are shown in the figure. (B) An Upset plot visualization of RF dataset-specific markers intersections across the three datasets. Each bar represents the number of dataset-specific markers in that category and the orange dots below the bar indicate their conservation across the datasets. For instance, 1st bar shows the 15 RF dataset-specific markers that are common in all three datasets.

We built RF models by training on the individual dataset from each geographic region with 10-fold cross-validations and identified 40 top-ranked species from each geographic region (more than one species could get the same rank) as a differential set of markers. Together, these markers included 119 unique species, and among those, 15 were found in all three geographic regions. Others were randomly distributed among the geographic regions (Fig. 4B, Data Set S3). Some of the RF-identified microbial markers with ranks below 20 in at least one geographic region are shown in Fig. 4A. For performance evaluation, we selected different sets of RF-identified differential taxa for each geographic region (RF dataset-specific markers) and a set of 58 RF dataset-specific differential markers common in 3 or 2 geographic regions (common RF-specific markers).

Overall, differential analysis using these three methods showed that both dysbiotic and healthy gut microbiota differ based on geographic location, and the three methods used in this analysis confirmed that the results are also sensitive to analysis methods.

### Identification of global biomarkers for CRC.

**FIG 5:**
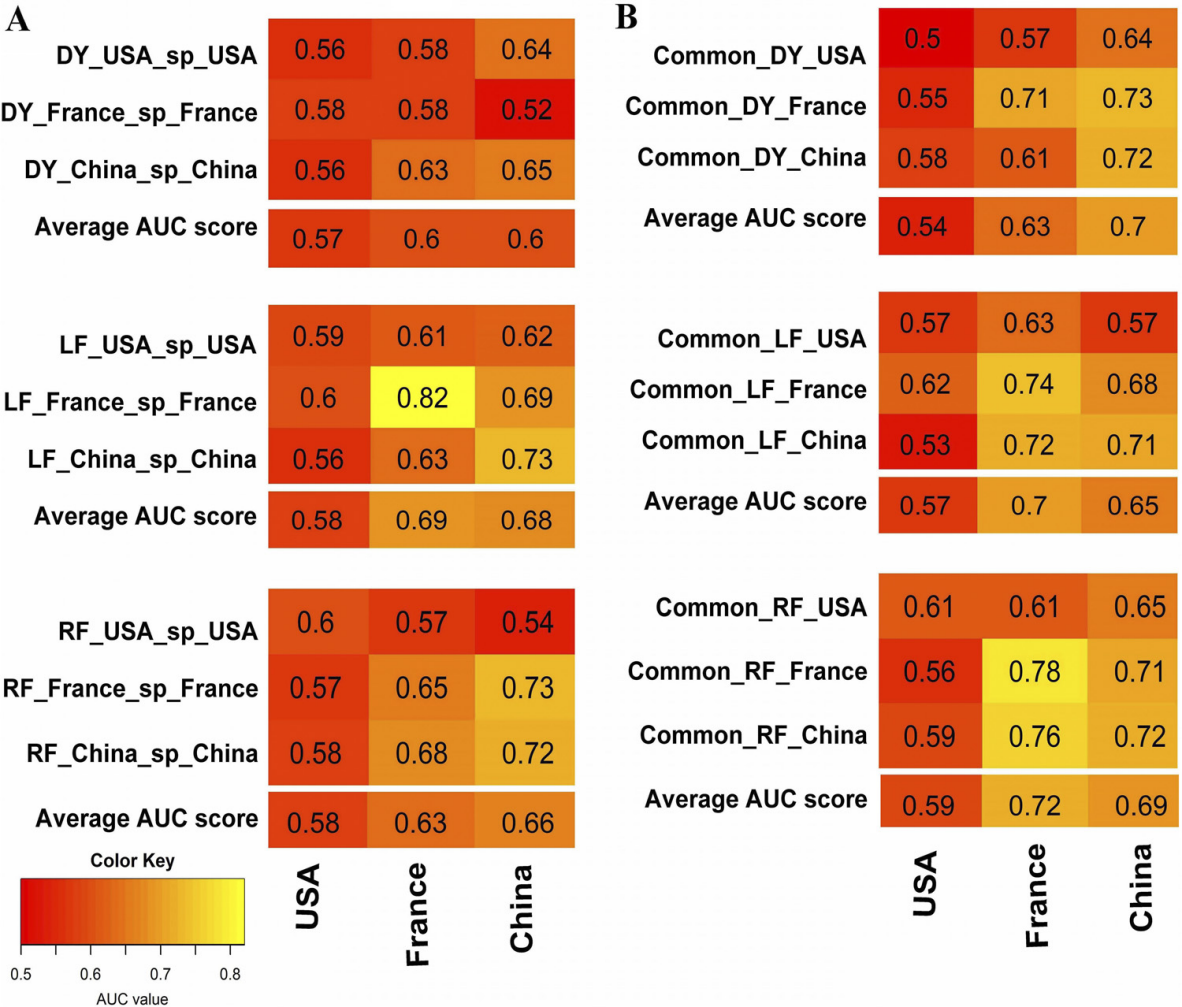
Prediction performance of the RF classifiers. Row indicates the RF classifier trained on the dataset-specific or common method-specific CRC markers; column indicates the classifier applying to the dataset of the corresponding column. In each three by three matrix of AUC values, diagonal values represent the AUC values of cross-validation obtained by using the trained row RF classifier on the column dataset, and off-diagonal values represent the AUC values of cross-cohort validation obtained by applying the trained row RF classifier on corresponding column dataset, (A) RF classifier was built from each dataset-specific markers (row). (B) RF classifier was built from the common markers present in at least two datasets from the USA, France, and China (common method-specific markers). ‘Average AUC score’ row represents the column average of the corresponding three-by-three AUC score matrix. Notation: e.g., DY_USA_sp_USA means classifier trained on the USA data based on the USA-specific markers identified by DyNet method, common_DY_USA means classifier trained on the USA data based on the common markers identified by DyNet method those are present in at least in two datasets DY, DyNet; LF, LEfSe; RF, random forest; and sp, specific.

RF classifiers were built based on the dataset-specific (markers identified from each dataset using three different methods described above) and common method-specific markers (combined dataset markers commonly found in at least two out of three datasets identified by the same method). To test the hypothesis that the set of CRC-associated taxa commonly present across different geographical regions could improve the prediction performance, we compared the performance score (AUC values) from the above classifiers, i.e., dataset-specific (Fig. 5A) and common method-specific (Fig. 5B). Results showed common DyNet-specific and common RF-specific markers performed better than the respective dataset-specific markers. The classifiers based on DyNet-identified markers in individual datasets (USA-, France- and China-specific markers) showed average performance scores ranging from 0.57 to 0.60 whereas classifiers based on common DyNet-specific markers' average performance scores ranged from 0.54 to 0.70 across the datasets. The dataset-specific markers identified by RF showed average performance ranging from 0.58 to 0.66, whereas common RF-specific markers average performance ranged from 0.59 to 0.72 across the datasets. Among the dataset-specific markers, LEfSe dataset-specific markers have average performance scores (0.58 to 069) across the geographic regions with a maximum value for the French population (0.82) and the average performance scores were nearly equal to average performance scores of common LEfSe-specific markers (0.57 to 0.70). Overall, the common method-specific markers performed better than dataset-specific markers. Among the common method-specific biomarkers classifiers, RF-specific classifiers showed the highest AUC scores in cross-validation and cross-cohort validation (range of AUC score 0.56 to 0.78), whereas LEfSe-specific markers showed AUC scores ranging from 0.53 and 0.74 and DyNet-specific markers showed performance values ranging from 0.50 to 0.73 on cross-validation of datasets. Hence, we considered common RF-specific markers for further validation on Austrian and Japanese datasets, which were not part of the training datasets.

**FIG 6:**
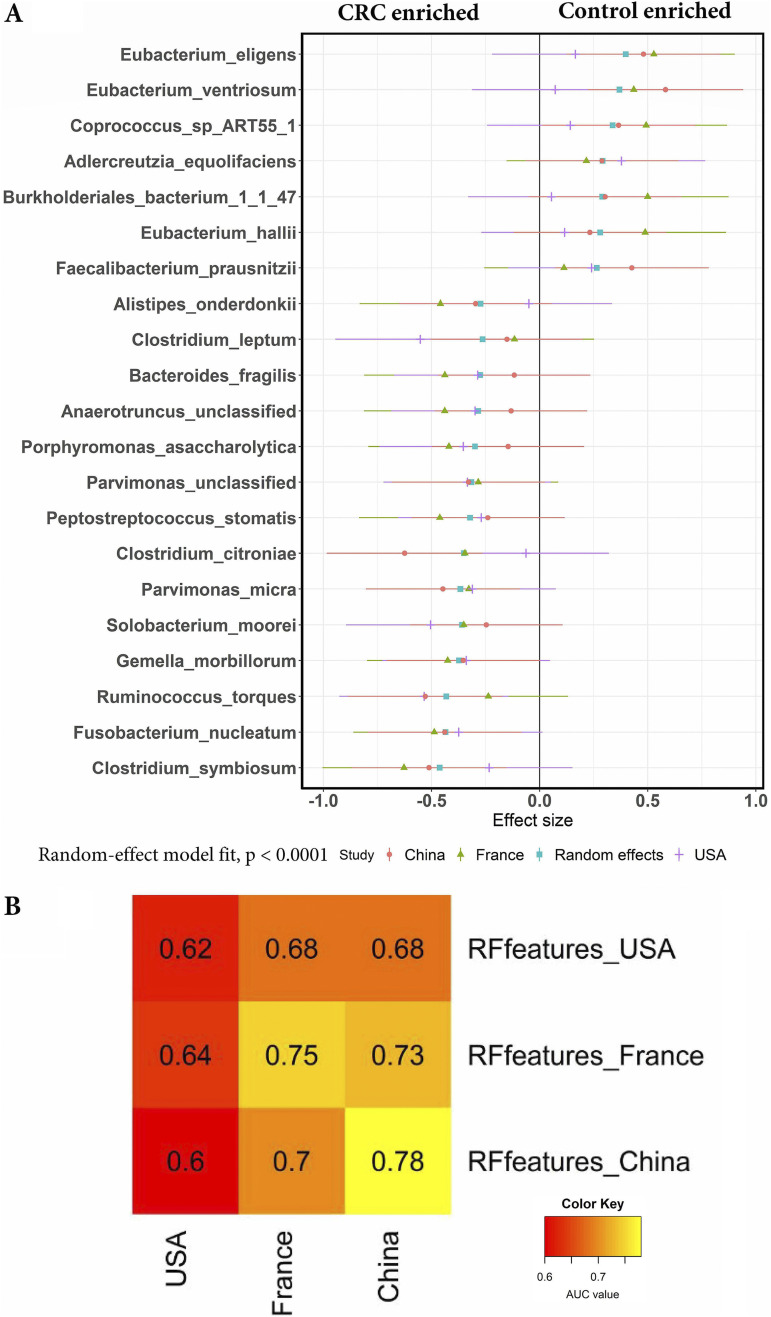
(A) Meta-analysis of selected RF CRC biomarkers markers using MetaPhlAn2 profiles from USA, China, and France geographic regions. The colored lines represent the 95% confidence interval for each dataset and random effect model estimate. (B) Cross-validations of a minimum set of RF CRC biomarkers on USA, France, and China datasets. The AUC values on each cell of the heatmap were obtained by the RF classifier (built from selected RF features) trained on the dataset row and applying the classifier on the dataset column.

The routine clinical procedures require a minimum set of diagnostic markers that are cost-effective and advantageous. For this purpose, we took 58 RF-specific markers and another 20 species having RF ranking below 30 in at least one dataset. Then calculated the random effect size of each marker based on standardized mean differences and selected the 21 biomarkers with the largest effect size, and are associated with CRC in all three datasets or associated with controls in all three datasets (Fig. 6A) and validated on the Japanese dataset (258 CRC cases and 246 healthy controls) and the Austrian dataset (46 CRC cases and 57 healthy controls). The small set of RF-specific markers (21 species) has similar performance compared with the larger set of RF-specific markers (58 species) on the USA, Chinese, and French datasets (cross-validation AUC score range from 0.62 to 0.78 AUC) (Fig. 6B) and cross-validation AUC scores on Austrian and Japanese datasets were 0.66 and 0.61. Among the 21 microbial markers, 14 species have the largest effect size in CRC samples, whereas 7 species have the largest effect size in control samples across the USA, China, and France regions (Random-effect model fit, *P* < 0.0001). The species associated with CRC include C. symbiosum, F. nucleatum, R. torque, G. morbillorum, S. moorei, P. micra, Clostridium citroniae, and others. In contrast, most of the nonpathogenic microbial species associated with controls include E. eligens, Eubacterium ventriosum, E. hallii, Bifidobacterium catenlatum, and others (Fig. 6A). Most of the CRC biomarkers reported in this study are consistent with previous studies, where F. nucleatum reported as an oral pathogen was shown enriched in CRC patients (28, 30–32), P. stomatis and *S. moorei* were reported as enriched in saliva and stool of CRC patients (33), P. micra, an obligate anaerobe was associated with CRC and E. ventriosum was shown to be associated with healthy controls (7), and B. fragilis was identified as a CRC biomarker in the previous studies (29, 34).

### Gene families of individual strains of selected microbial species.

**FIG 7:**
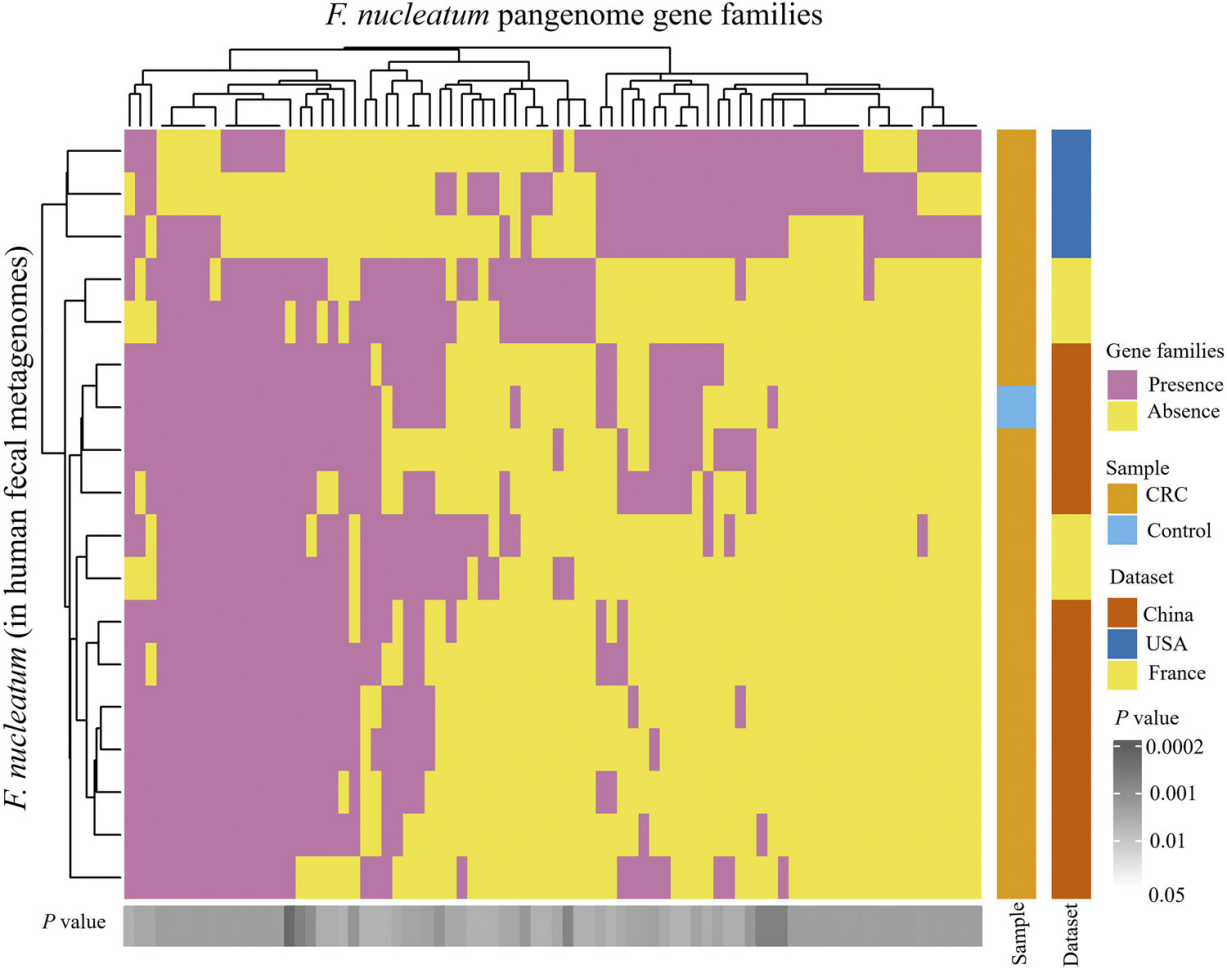
Heat maps of the strain-level genomic diversity of F. nucleatum across three geographic regions: USA, China, and France. The significant differential gene families (*P* < 0.05) were identified using Fisher's exact test on presence and absence gene family profiles.

Strain-level gene families of microbial species were investigated to better understand the genomic differences of CRC pathogens across geographic regions using PanPhlAn software. For this analysis, we considered CRC-associated microbes, F. nucleatum, and B. fragilis and analyzed the strain-specific genes present in 346 (180 CRC cases and 166 controls) metagenomes from the USA, French and Chinese populations. F. nucleatum was found in 74 CRC and 11 control metagenomes, and its strain-level profiles were identified based on its 15,239 pangenome gene families, whereas B. fragilis was found in 149 CRC and 110 control metagenomes, and its strain-level gene-families were identified based on its 29, 335 pangenome gene-families. Individual strains of F. nucleatum were detected in 11 metagenomes (10 CRC and 1 control) (Data Set S4). Statistical analysis of gene families showed significant differences in 80 gene families across F. nucleatum strains of USA, Chinese, and French populations (*P* < 0.05) (Fig. 7). The F. nucleatum strains of the USA were separated from those of the French and Chinese populations, whereas few strains from the French population were similar to strains from the Chinese population. Further mapping with the UniProt database showed that LPS core biosynthesis, adenosylcobalamin biosynthesis, NAD (+) biosynthesis, isoprenoid biosynthesis, phospholipid metabolism, and tRNA modification pathways were significantly different across the strains of three geographical regions. PanPhlAn analysis of B. fragilis identified strains in 32 metagenomic samples (23 CRC and 9 controls) (Data Set S4), and 605 gene families were significantly different in the strains from the USA, French, and Chinese populations (*P* < 0.05) (Fig. S10). Mapping with the UniProt database showed the functional differences in gene families related to choloylglycine hydrolase, TonB-dependent receptors, OmpA/MotB domain proteins, histidine kinase, BfmA, and ArsR family transcriptional regulator proteins.

### Predicted metabolome changes associated with CRC.

**FIG 8:**
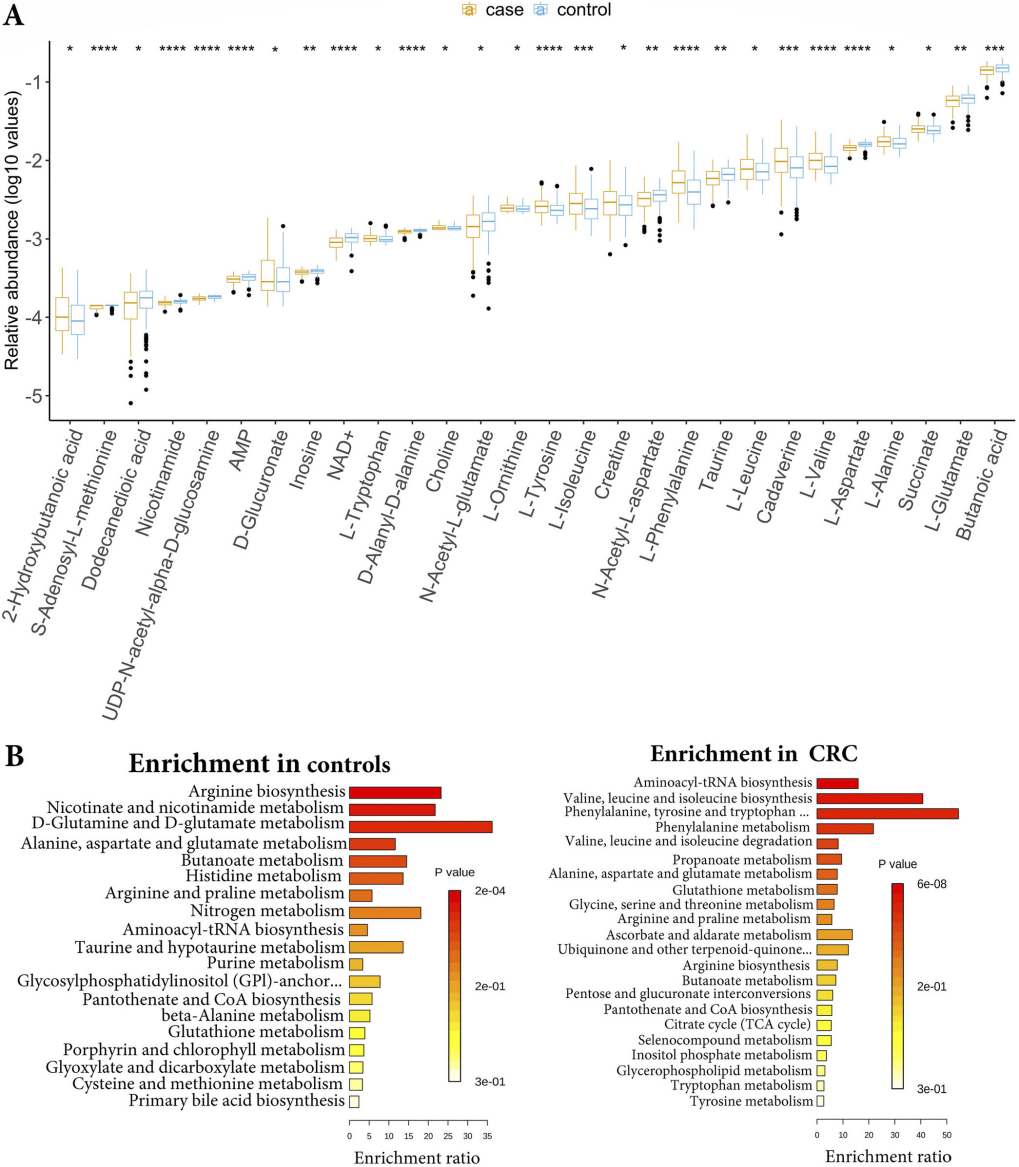
Analysis of MelonnPan predicted metabolites from the USA, China, and France datasets. (A) The relative abundance of significantly different (*P* < 0.05) metabolites between CRC and healthy control groups. Blue indicates the control samples and red indicates the CRC samples from all three datasets. Enrichment of pathways based on predicted metabolites in (B) healthy controls and (C) CRC samples.

The metabolite profiles of the gut microbial communities from the USA, French, and Chinese populations were predicted using *MelonnPan* software. This analysis predicted 135 metabolites from each dataset. The predicted metabolite profiles were filtered to remove <0.01% relative abundance in ≥10% of the samples as suggested by the developers of the *MelonnPan* software (35). After filtering we were left with 70 metabolites in each dataset. The filtered metabolite profiles of all datasets were merged and normalized and analyzed using *edgeR* and *limma* software. Differential analysis showed significant changes in the metabolite profiles between CRC and control samples (*limma*, *P* < 0.05; Fig. S11 and Data Set S4). Amino acids, including phenylalanine, valine, leucine, alanine, isoleucine, and tyrosine, and other metabolites, including cadaverine, succinate, and creatine, were highly enriched in CRC, whereas butanoic acid, l-glutamate, and l-aspartate were abundant in the controls (Fig. 8A). Similarly, pathway enrichment analysis of differential metabolites showed pathways related to amino acid metabolism enriched in CRC (Fig. 8C), whereas arginine biosynthesis, nicotinate, and nicotinamide metabolism, d-glutamine, and d-glutamate metabolism, and butanoate metabolism were enriched in controls (*P* < 0.05, Fig. 8B). The metabolite profiles and pathway associations in CRC indicate altered energy sources required for the high metabolic growth rates of CRC cells. In addition to glucose, the cancer cells use amino acids like glutamine, valine, leucine, and isoleucine as alternate energy sources to meet the high energy demand for growth and as biosynthetic molecules required for tumor growth (36).

**FIG 9:**
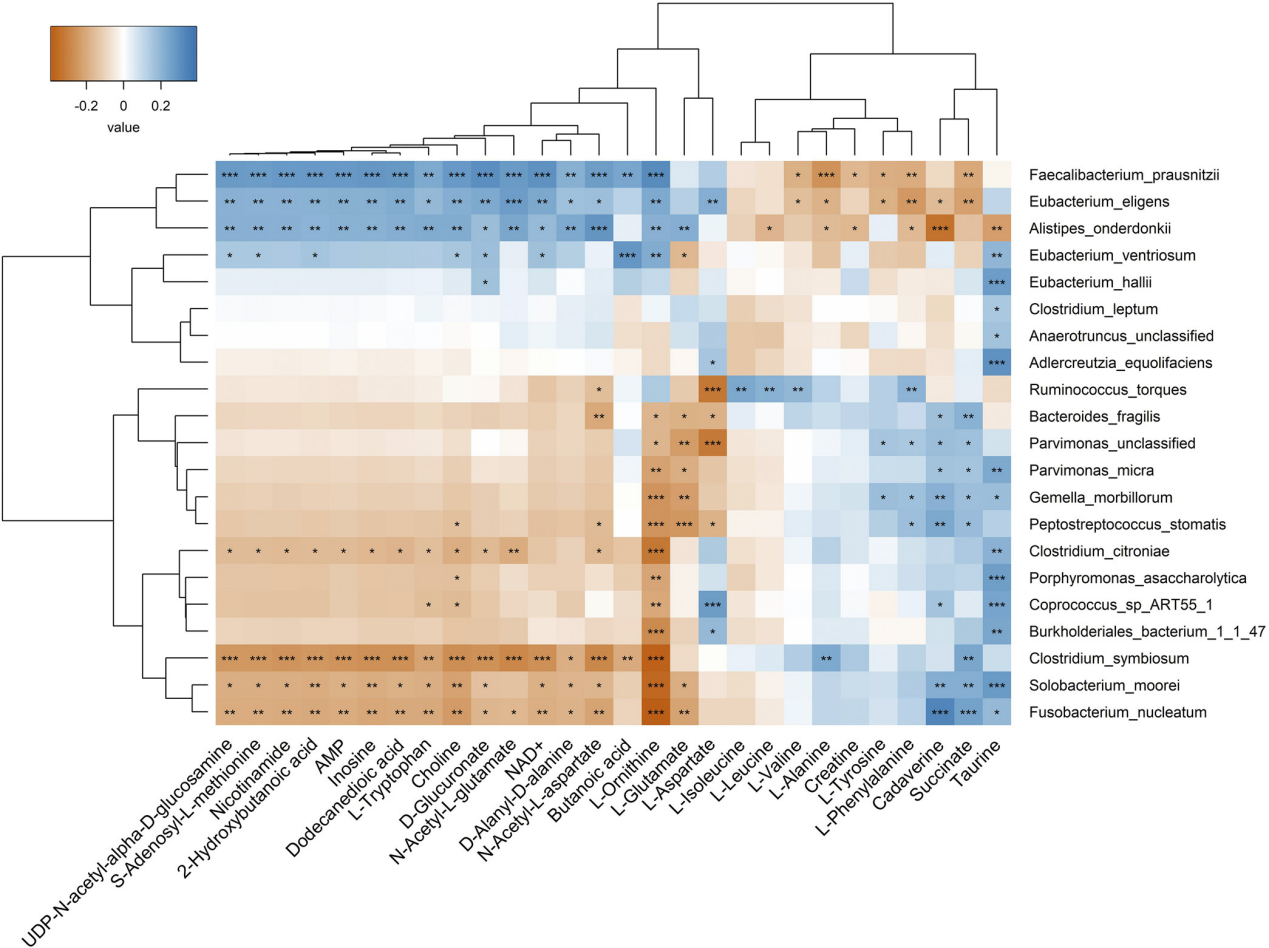
Correlation analysis between 21 CRC microbial markers and 28 metabolites that were significantly different between CRC and healthy gut communities, 14 were enriched in CRC cases and 14 were enriched in healthy controls. Red indicates the positive correlation and blue indicates the negative correlation. *, *P* ≤ 0.05; **, *P* ≤ 0.01; ***, *P* ≤ 0.001.

We also estimated the correlations between gut microorganisms and metabolites in CRC samples from three geographic regions USA, France, and China using Spearman correlation and examined the correlations between differential CRC microbial biomarkers and metabolites identified in our study. The heatmap (Fig. 9) showed the separation of CRC microbial markers into clusters (vertical axis). The top clusters are mostly control-associated organisms like F. prausnitzii, E. eligens, E. ventriosum, and E. halli and the bottom clusters are CRC-associated such as F. nucleatum, S. moorei, and C. symbiosum in one cluster and R. torques, B. fragilis, *Parvimonas* spp, G. morbillorum, and P. stomatis into another cluster. Even though Alistipes onderdonkii, a CRC-associated microorganism, was clustered with F. prausnitzii and E. eligens (control-associated), they differed in correlations with butanoic acid, glutamate, aspartate, and tyrosine. However, the CRC-associated microorganisms, C. citroniae, and P. asaccharolytica grouped with control-associated microorganisms like *Coprococcus* sp. *ART55_1* and Burkholderiales bacterium *1_1_47* differed in correlations with aspartate.

Metabolites such as cadaverine, succinate, and phenylalanine were enriched in CRC samples and showed significant positive correlations with most CRC-associated microbes like P. stomatis, G. morbillorum, B. fragilis, *Parvimonas* spp., F. nucleatum, S. moorei, and C. symbiosum, while showing significant negative correlations with control associated microbes, F. prausnitzii, and E. eligens (*P* < 0.05). Branched-chain amino acids, including valine, leucine, and isoleucine, were positively correlated with the presence of R. torques. Butyrate, a short-chain fatty acid metabolite, was positively associated with F. prausnitzii and E. ventriosum, and negatively associated with C. symbiosum (*P* < 0.05). Alanine was enriched in CRC samples and showed a significant positive correlation with C. symbiosum, while taurine was positively correlated with both CRC- and control-associated gut microorganisms. Ornithine showed significant positive correlations with most of the control-associated microbes and negative associations with CRC pathogens (*P* < 0.05) (Fig. 9, Data Set S4). Ornithine is an important metabolite in the arginine metabolism produced by lactobacilli, which helps in gut mucosal barrier function (37). A major cluster of metabolites such as nicotinamide, choline, tryptophan, 2-hydroxybutanoic acid, and others showed positive correlations with control-associated microbes, F. prausnitzii and E. eligenes (top cluster) whereas negatively correlated with F. nucleatum, S. moorei, and C. symbiosum (bottom cluster). CRC-associated bacteria, A. onderdonkii showed positive correlations with most of these metabolites except butanoic acid and aspartate (*P* < 0.05).

## DISCUSSION

Various studies were focused on the dysbiotic gut microbiota to identify specific microbial associations as etiological agents of CRC (22, 23, 38, 39), but the results were inconsistent across the studies. Because the composition of the gut microbiome varies with different environmental stimuli, dietary habits, and genetic traits, the independent geographic region/cohort-specific dataset analysis would result in biased associations related to dominant factors that cannot be generalized. In this study, we carried out a meta-analysis of multiple datasets from diverse geographic regions and identified a set of global CRC biomarkers associated with all datasets. By selecting common CRC-microbial associations across geographic regions, we can avoid nonspecific associations introduced by the heterogeneity factors. Our results showed that the RF method performed better than the other methods, followed by LEfSe and co-occurrence network methods. RF-specific markers showed a consistent increase in performance from dataset-specific markers to common method-specific markers, whereas the LEfSe method performed better with independent dataset-specific biomarkers than using common LEfSe-specific biomarkers. Co-occurrence networks are often used to examine microbial associations among key taxa in ecological communities. A recent microbiome study used this approach to identify the key taxa associated with acute pulmonary exacerbations (40). In our study, we implemented a co-occurrence network for the first time to identify and compare CRC biomarkers from the rewired nodes between CRC and control networks. Rewired nodes across the networks indicate altered interactions among the community members. In our analysis, co-occurrence network-specific features showed a lower performance score compared to the other two methods. However, it helped to identify significant interactions between oral pathogens in CRC and a healthy gut, and these results are consistent with the previous study (28). RF was one of the most widely used methods for the identification of biomarkers in microbiome studies. In our study, RF models showed a consistent increase in performance from RF dataset-specific markers to common RF-specific markers. Due to this reason, we selected the global CRC markers identified by the RF method.

Most of the CRC biomarkers that we identified in this study are consistent with the previous investigations (39, 41). We identified a new CRC biomarker, A. onderdonkii. The genus *Alistipes* is generally considered a commensal organism. However, it may contribute to inflammatory effects in the host under dysbiotic conditions due to the presence of putrefaction pathways such as histidine degradation and production of tetrahydrofolate, indole, and phenol (42). Similarly, we also identified C. citroniae as a biomarker, it belongs to the newly defined *Lachnoclostridium* genus. It has been reported in early stage to later stage CRC samples with incremental abundances (43). On the other hand, the control-associated biomarkers are gut commensal organisms, which include F. prausnitzii, E. ventriosum, and E. hallii. They are known to produce SCFAs and regulate gut mucosal health (44).

*PanPhlAn* analysis of CRC-associated microbe, F. nucleatum, showed a significant difference in gene families related to isoprenoid biosynthesis and phospholipid metabolism. These pathways are involved in lipid metabolism, and dysregulation of lipid metabolism was identified as a characteristic feature and correlated with shorter survival rates in CRC (45, 46). Similarly, other studies showed increased phospholipids were observed in colonic neoplasms (47) and upregulation of the mevalonate-isoprenoid (MIB) pathway was identified in CRC stem cells (48). Enterotoxigenic B. fragilis has been shown to contribute to colon carcinogenesis, and its pathogenicity is mainly due to its capsule, outer membrane proteins, and a metalloprotease protein toxin (B. fragilis toxin) (49). In this study, we identified that the B. fragilis strains present in the USA, Chinese, and French populations mainly differed in the gene families related to TonB-dependent receptor, OmpA/MotB domain protein, BfmA, choloylglycine hydrolase, MobA, multidrug efflux MFS transporter, type IV secretion system needle protein Hcp and other putative proteins. Most of these proteins play important roles in bacterial pathogenicity like toxin transport, colonization, biofilm formation, nutrient uptake, and multidrug resistance and are produced from bacterial pathogenicity islands (50). The type IV secretion system is involved in the transfer of virulence factors to the host and TonB-dependent receptors and OmpA are involved in the uptake of nutrients and other small molecules into the cell (51). Choloylglycine hydrolase is a conjugated bile salt hydrolase known to be produced by gut microbes (strains from Bacteroidetes) that has the potential to alter the host fatty acid metabolism by mediating bile salt hydrolysis (52). In strain-level metagenomic analysis, both F. nucleatum and B. fragilis strains showed differences in gene families that can affect the host fatty acid metabolism. This indicates the crosstalk between the gut microbes and host metabolism through microbial metabolites in CRC.

The metabolite analysis showed enrichment of SCFAs such as butanoic acid in control samples than CRC samples and positively correlated with control-associated microbes. It is one of the by-products of the fermentation of fiber by gut microbiota, that is linked to gut homeostasis and the prevention of tumor growth (26, 53). Succinate, one of the intermediates in the tricarboxylic acid (TCA) cycle was enriched in CRC samples and positively correlated with CRC-associated microbes. It is also known to be produced by gut microbes such as B. fragilis, F. prausnitzii, *Alistipes* spp., *Prevotellaceae*, and others. Gut microbial dysbiosis can lead to the accumulation of succinate and intestinal inflammation (54). Both CRC- and control-associated gut microorganisms showed a positive correlation with taurine. Meat-rich diets promote taurine conjugation, which leads to increased taurocholic acid formation in the small intestine. Later the deconjugation of taurocholic acid by diverse gut bacteria (in the large intestine) generates cholic acids and taurine, which in turn are converted to carcinogenic secondary bile acid, deoxycholic acid, and a cytotoxic compound, hydrogen sulfide, respectively (55).

Interestingly amino acids valine, leucine, isoleucine, and phenylalanine showed enrichment in CRC than controls, which has been previously observed in CRC (56, 57). Glutamate was enriched in control samples compared to CRC samples, which contrasts with previous studies (57) that showed various interactions with both CRC- and control-associated microbes. Polyamine-cadaverine was enriched in CRC and positively associated with CRC-associated microbes, consistent with a previous study (56). Polyamines are essential for normal cell growth, they are produced by the host, gut bacteria, and dietary origin, however, dysregulation of polyamine metabolism is linked to colon cancer (58). Choline was enriched in CRC which is consistent with a previous study by Thomas et al. (41) where they showed an abundance of choline degradation genes (*cutC* and *cutD*) in the CRC gut microbiome. However, in our study, choline is positively correlated with control-associated microbes. Overall, our study provided a reliable set of global microbial biomarkers for CRC identification that can be used across different populations. Moreover, we reported the differentiating metabolites and important gut microbe-metabolite interactions in CRC, which may have the potential to influence host metabolism. This study would pave the way for further investigations that could lead to the development of noninvasive diagnostic tools and therapeutic interventions for CRC management.

## MATERIALS AND METHODS

Fecal shotgun metagenomics sequencing datasets of CRC patients and healthy controls belonging to three different geographical regions USA (24), China (7), and France (23) were downloaded from the public database, the European Nucleotide Archive (ENA). The other two fecal shotgun datasets of CRC and control samples from Austria (22) and Japan (38) were downloaded from ENA and DDBJ Sequence Read Archive (DRA), respectively, for validation purposes. Details of the samples with accession numbers of the datasets used in this study are provided in Table S1, Metadata for USA, China, France, and Austria datasets were obtained as JSON-formatted files from EBI BioSamples and parsed using Perl/Python scripts into tables with different meta fields that include sample ID, study ID, secondary ID, sequencing type, country, age, BMI, and diagnosis. For the Japanese dataset, metadata was obtained from the original publication (38). For metabolomic profiling of gut communities, the metabolomic profiles of the Japan dataset were obtained from the original work by Yachda and group (38), and the list of 250 samples of the Japan dataset used in this analysis is in Data Set S4.

### Taxonomic profiling of metagenomic datasets.

Metagenomic sequencing datasets obtained from all five geographical regions were quality filtered using *FastQC* software and aligned against Coliphage phi-X174 (PhiX) and GRCh38 human reference genomes to remove PhiX and human read contaminations using *BBduk* and *BBmap* tools (59). After preprocessing, samples from all datasets were quantified for taxonomic composition using *MetaPhlAn2* (60) software. *MetaPhlAn2* relies on unique clade-specific markers identified from about 17,000 reference genomes from bacterial, archaeal, viral, and eukaryotic microorganisms for microbial profiling and quantification. In this study, we used species-level taxonomic profiles in all analysis methods. The taxonomic profiles of each dataset (each dataset represents a geographic region considered in this study) were filtered to remove species present at a relative abundance value of < 0.1% across the samples to reduce potential false-positives (61). For the downstream analyses, the species abundance of each dataset was mapped with related metadata using the *Phyloseq* package in R. Alpha and beta diversity indices of the gut microbiome for each dataset were estimated based on the relative abundance profiles of species using the *Vegan* package in R and plotted using the *ggplot2* package in R.

### Identification of microbes specific to CRC or healthy gut communities.

Statistical, machine learning, and network-based methods were used in this study to identify CRC biomarkers. To describe briefly, linear discriminant analysis (LDA) effect size (*LEfSe*) is a statistical method to identify key taxa that are significantly different between CRC cases and controls. Species-level relative abundance data along with sample details were analyzed separately for each dataset using *LEfSe* software (62). It uses nonparametric tests to identify significant features, performs subclass comparisons, and then LDA to estimate the effect size of identified features. The differential taxa for each dataset were considered based on the Effect size LDA score > 2 and FDR-adjusted *P* ≤ 0.05, (ii) RF algorithm is the most used machine learning method on microbiome data to identify differential microbial features (41). The random forest and caret packages in R were used for the RF model building. Standardized relative abundance data (Z-score) of microbial species in all samples and group information were used as input for RF analysis. RF classifiers were built by training on each dataset separately with 10-fold cross-validation. RF features with a higher mean decrease in the Gini index (top-ranked microbial species) from each dataset were considered differential markers. The performance of each classifier was evaluated using 10-fold cross-validation and AUC metrics, (iii) Co-occurrence networks for CRC cases and controls were inferred separately for each dataset based on the correlation coefficients calculated using *SparCC* v 0.1 software (63) from the species-level relative abundance data (normalized to 1 million counts) of corresponding datasets. *SparCC* estimates the Pearson correlation between species from the log-ratio transformation values while accounting for the compositionality of metagenomics data (64). The correlation coefficients were estimated by the permutation-based approach in *SparCC*, and the Pseudo *P* values were calculated for the bootstrapped correlation coefficients. Network plots were constructed considering significant co-occurrence correlations (FDR < 0.05) in *Cytoscape* 3.7.1 (65). Then, the highly connected dense regions in the network were detected using *the MCODE* plugin. The CRC and control networks of each dataset were synchronized using the *DyNet* plugin (66) and then identified the most rewired nodes across the two network states. The highly rewired nodes between CRC and control networks (rewiring metric or D_n_-score ≥2.0 and an edge count ≥4) from each dataset were considered differential taxa for CRC.

### Assessment of predicted biomarkers.

Differential taxa from each method were tested among the datasets using RF models with 10-fold cross-validations. The performance of each classifier was measured in terms of the AUC metric and selected biomarkers with high AUC scores. The random effect size of each selected biomarker was calculated based on standardized mean differences using the *Metafor* package (67) in R. Biomarkers with the highest random effect size associated with either CRC or control groups were selected as CRC global biomarkers and further analyzed for metabolite interactions in CRC gut communities.

### Strain-level metagenomic profiling of CRC pathogens.

The gene composition of individual strains of CRC-associated microbe in CRC and healthy gut metagenomes from the USA, China, and France were identified using *PanPhlAn* v 3.0 software (68). *PanPhAn* identifies the gene presence and absence within different strains of species in metagenomes based on the entire set of the species’ pangenomes. Differential analysis of gene families was performed across the geographical regions using Fisher’s exact test (fisher.test() in R). Then significant differential gene families were mapped to UniProt Knowledgebase (69) to understand their functional roles in CRC.

### Metabolomic profiling of gut communities.

Metabolomic profiles of CRC and healthy gut metagenomes were predicted using *MelonnPan* v1.0.0 software (35). In brief, *MelonnPan* builds a model based on paired metabolomic and metagenomics features of a community and uses that model to predict the metabolite relative abundances for a given metagenomic community based on its set of features derived from sequencing data. Paired microbial and metabolite relative abundance data of the Japanese CRC dataset (250 samples listed in Data Set S4) were used to build a predictable model and predicted the metabolite profiles of the USA, China, and France datasets based on species relative abundances. Predicted metabolomic profiles were combined and analyzed using the *edgeR* and *limma* package in R (70, 71), and the significant differential metabolites were analyzed for pathways using *MetaboAnalyst* v5.0 (72). Correlations between differential metabolites and CRC microbial biomarkers were estimated using the Spearman correlation method and results were plotted using *ggplot2* and *gplot* packages in R.

### Statistical analyses.

Nonparametric Fisher’s exact test was used for the differential analysis of gene families. Microbe-microbe or microbes-metabolites correlations were estimated using Spearman’s correlation coefficients. For statistical significance *P* < 0.05 and Benjamini-Hochberg corrected FDR values were considered appropriate. Standardized (Z score) data was used for metabolite analyses. The covariate effect on species diversity was tested using a multivariate linear regression method. The batch effect on metabolite profiles was tested using *edgeR* and *limma* in R. All data analyses and visualizations were conducted in R v 4.0.0. and above (73).

### Data availability.

The original contributions presented in the study are included in the article/Supplemental Material. Further inquiries can be directed to the corresponding author.
