# Supplementary material for: Meta-Analysis of Altered Gut Microbiota Reveals Microbial and Metabolic Biomarkers for Colorectal Cancer
Source: Microbiol Spectr. 2022 Jun 29;10(4):e00013-22. doi: 10.1128/spectrum.00013-22 (PMC9431300; doi:10.1128/spectrum.00013-22)
Supplement: Supplemental file 5 — Table S1 and Fig. S1 to S11. Download spectrum.00013-22-s0001.pdf, PDF file, 4 MB [file spectrum.00013-22-s0001.pdf]

Supplementary data

Dataset-geographic regions (Data source)	Sequence accession codes	Samples used in this study	Age (Average±s.d.)	BMI (Average±s.d.)	Sex F(%)/M(%)
USA(ENA)	PRJEB12449	52 CRC	61.84±13.58	24.88±4.24	28.84/71.15
		52 Control	61.23±11.02	25.34±4.27	28.84/71.15
France (ENA)	ERP005534	53 CRC	66.81±10.87	25.56±5.17	45.28/54.71
		61 Control	60.57±11.38	24.67±3.17	54.1/45.9
China (ENA)	PRJEB10878	74 CRC	65.93±10.56	23.95±3.15	35.13/64.86
		54 Control	61.83±5.69	23.5±2.97	38.88/61.11
Austria (ENA)	ERP008729	46 CRC	67.06±10.91	26.5±3.52	39.13/60.86
		61 Control*	66.96±6.45	27.61±3.77	40.98/59.01
Japan (DDBJ)	DRA006684, DRA008156	258 CRC	62.72±9.63	23.03±3.27	37.59/62.40
		246 Control	60.96±12.50	22.66±3.05	46.74/53.25

Supplementary Table 1: Details of the datasets and samples used in this study. CRC biomarkers were identified using the control and CRC samples belonging to the USA, China, and France geographic regions and validated using Japanese and Austrian's control and CRC samples. From the Japan dataset, we have used CRC and controls, it has other samples belonging to the history of colorectal surgery and a few polyps. The CRC samples from Japan dataset include stages 0, I, II, III, and IV. * 61 control samples were used in this analysis from the Austria dataset due to metadata issues (the original samples reported were 63 controls in the Austria dataset). ENA-European Nucleotide Archive, DDBJ- DNA databank of Japan database, F- female and M- male.

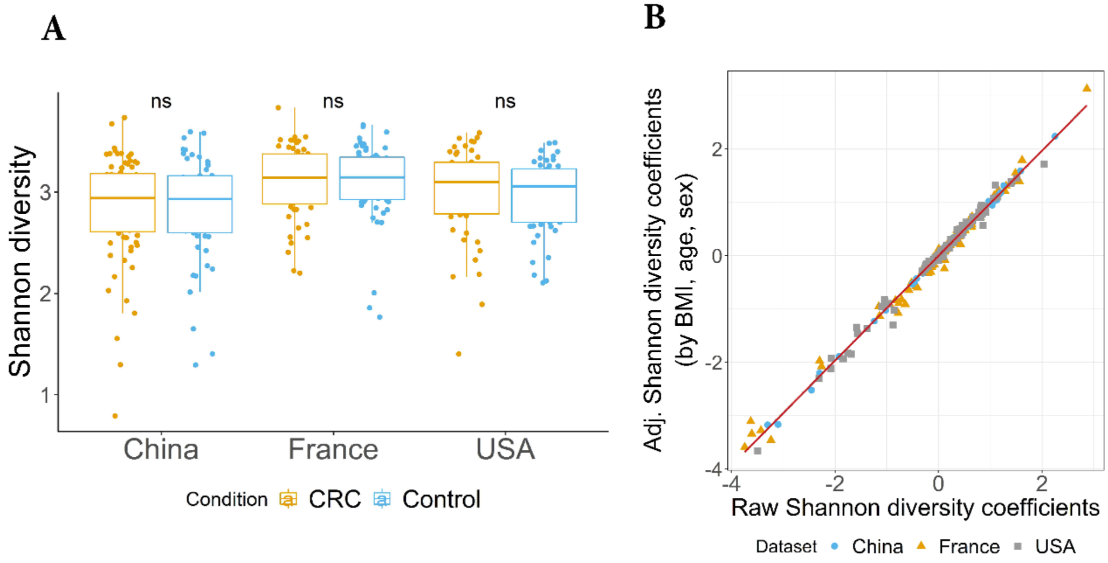

Supplementary Figure 1: Alpha diversity and covariate analysis among CRC and control groups. Analyses were performed at species-level taxa. (A) Boxplots showing the Shannon species diversity in each dataset. Differences between the groups were calculated by two-tailed Wilcoxon rank-sum tests; (B) Multivariate analysis of Shannon diversity coefficients using raw and BMI-, age- and sex-adjusted coefficients from linear regression models. ns- not significant where $P > 0.05$.

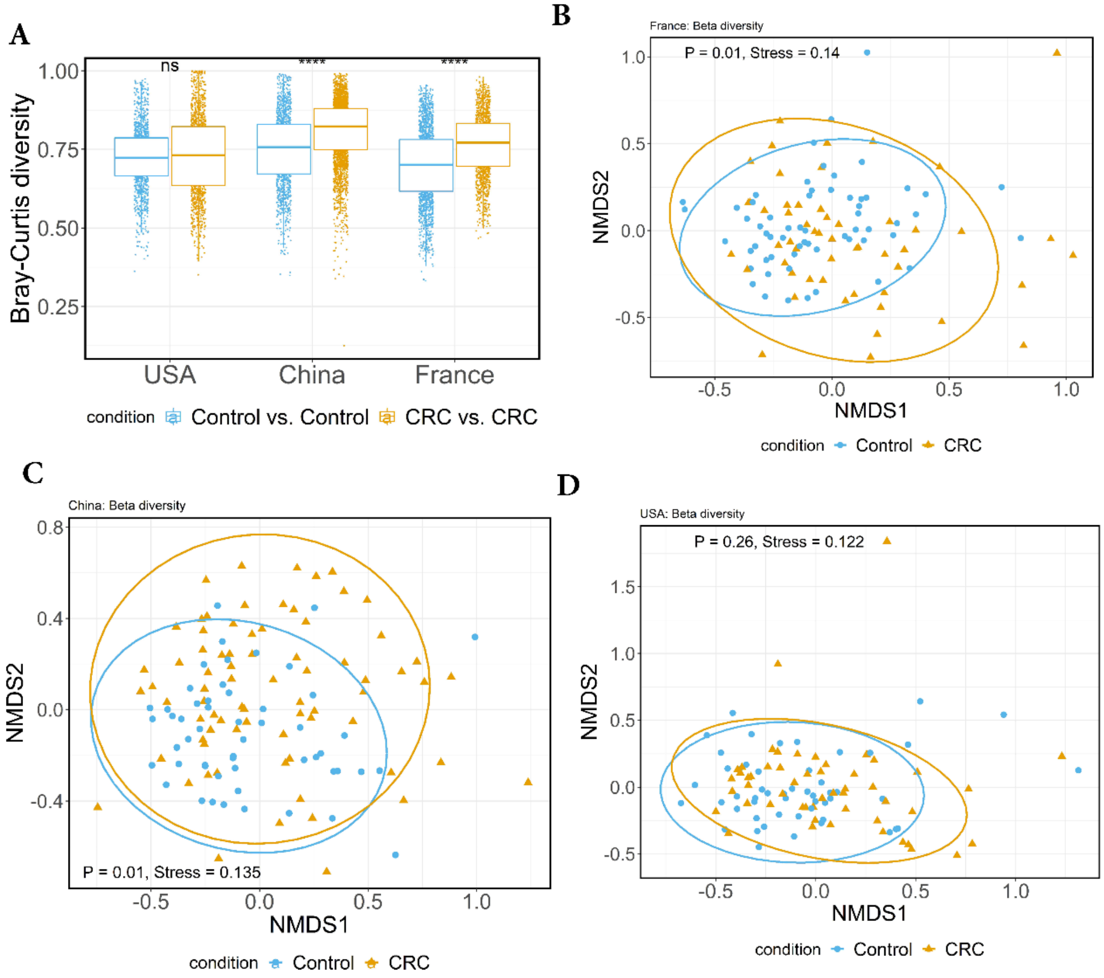

Supplementary Figure 2: Beta diversity comparisons between CRC and control groups. Analyses were performed at species-level taxa. (A) Boxplots showing the Bray-Curtis distances between the comparison pairs of gut microbial communities. Dots represent the distance between the samples in each comparison group and the lines in the boxes correspond to the median. Differences between the groups were calculated by two-tailed Wilcoxon rank-sum tests. ns- not significant where $P > 0.05$, * $P \leq 0.05$; **$P \leq 0.01$; ***$P \leq 0.001$. Non-metric Multidimensional Scaling (NMDS) ordination plots, (B) in the France dataset, (C) in China dataset, and (D) in the USA dataset. NMDS plots depict the beta-diversity among CRC and control groups, which were measured in Bray-Curtis dissimilarity metrics. Ellipses in the figure show the 95% confidence

intervals of each group of samples. Each dot denotes the microbial composition of a single sample in a low-dimensional space. Differences between the groups were measured based on the Adonis test.

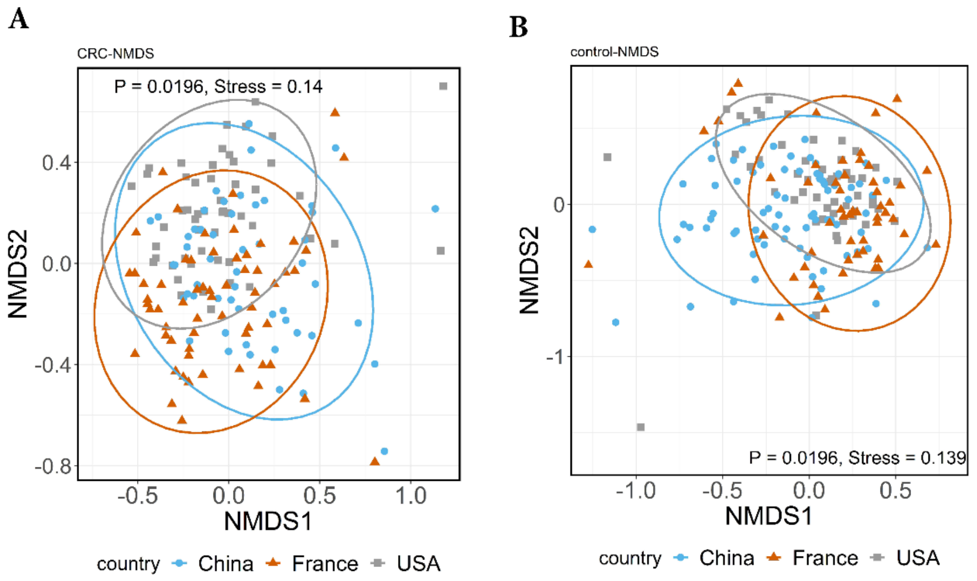

Supplementary Figure 3: Beta diversity among CRC and control groups. Analyses were performed at species-level taxa. Non-metric Multidimensional Scaling (NMDS) ordination plots depict the beta-diversity across the datasets. Ellipses in the figure show the 95% confidence intervals of each group of samples. Each dot denotes the microbial composition of a single sample in a low-dimensional space. (A) NDMS ordination in CRC samples across three datasets. (B) NDMS ordination in control samples across three datasets; The significance between the groups was measured based on the Adonis test.

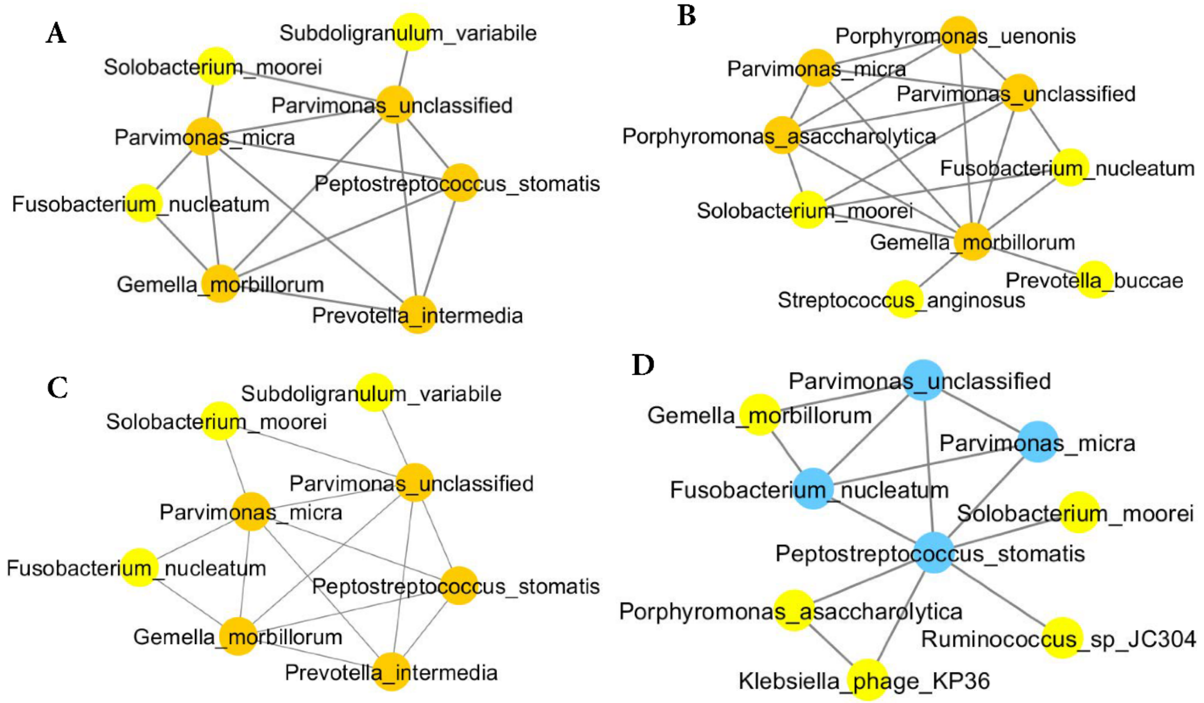

Supplementary Figure 4: MCODE clusters (in red nodes) with their first neighbors (yellow) nodes formed from oral pathogens in CRC and control co-occurrence networks of three datasets, (A) in the CRC network of France dataset, (B) in the CRC network of the USA dataset, (C) in the CRC network of China dataset, and (D) in the control network of China dataset.

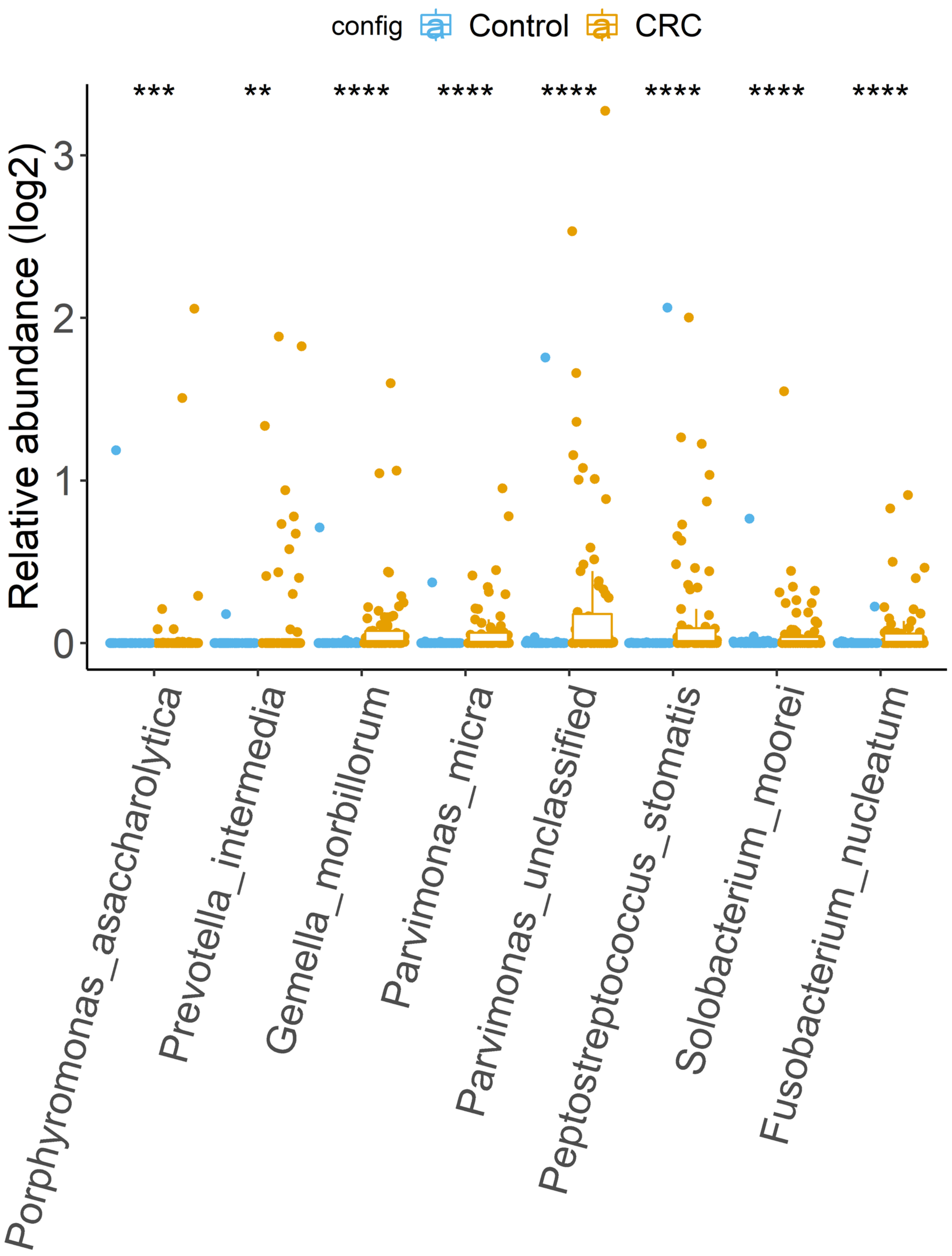

Supplementary Figure 5: Differential analysis of oral pathogens present in the CRC and control cooccurrence network cluster from the China dataset.

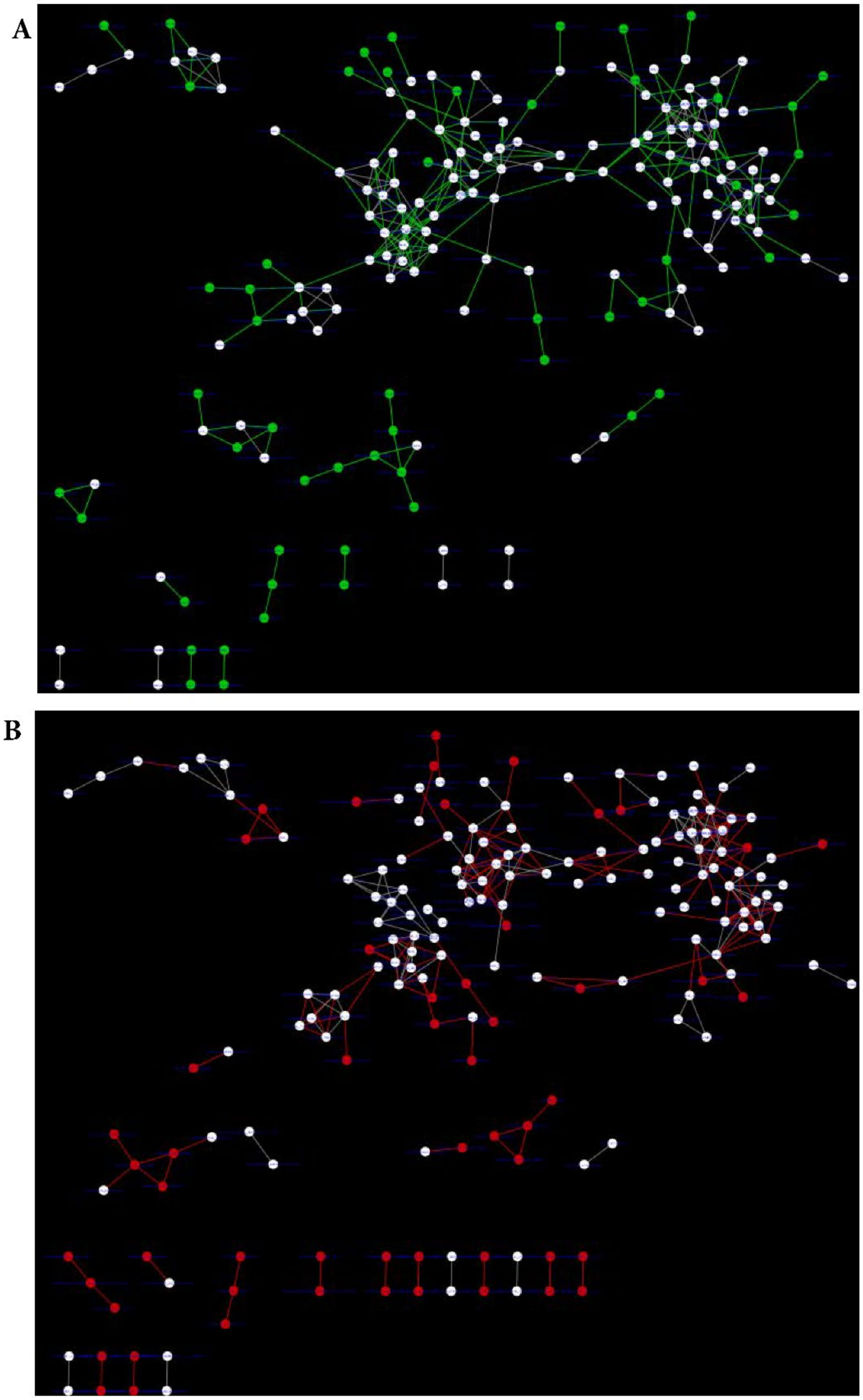

Supplementary Figure 6: DyNet visualization of co-occurrence networks of CRC and controls at the species level from the China dataset, (A) control network with unique green nodes and edges, and (B) CRC network with unique red nodes and edges, whereas the white-colored nodes are common in both networks.

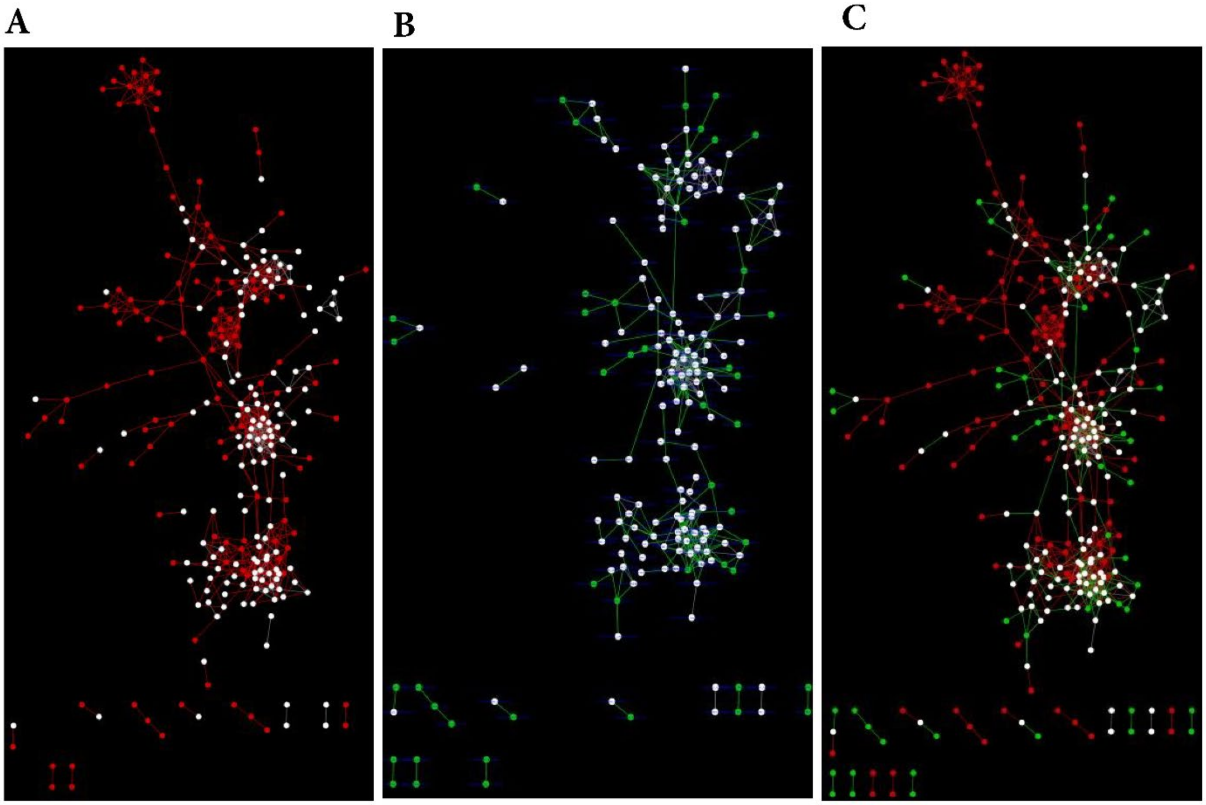

Supplementary Figure 7: DyNet visualization of co-occurrence networks of CRC and controls at the species level from the USA dataset, (A) CRC network, (B) control network, and (C) synchronized CRC and control co-occurrence networks. Red nodes and red edges are present only in the CRC network, green nodes and green edges are present only in the control network, whereas white nodes are common in both networks.

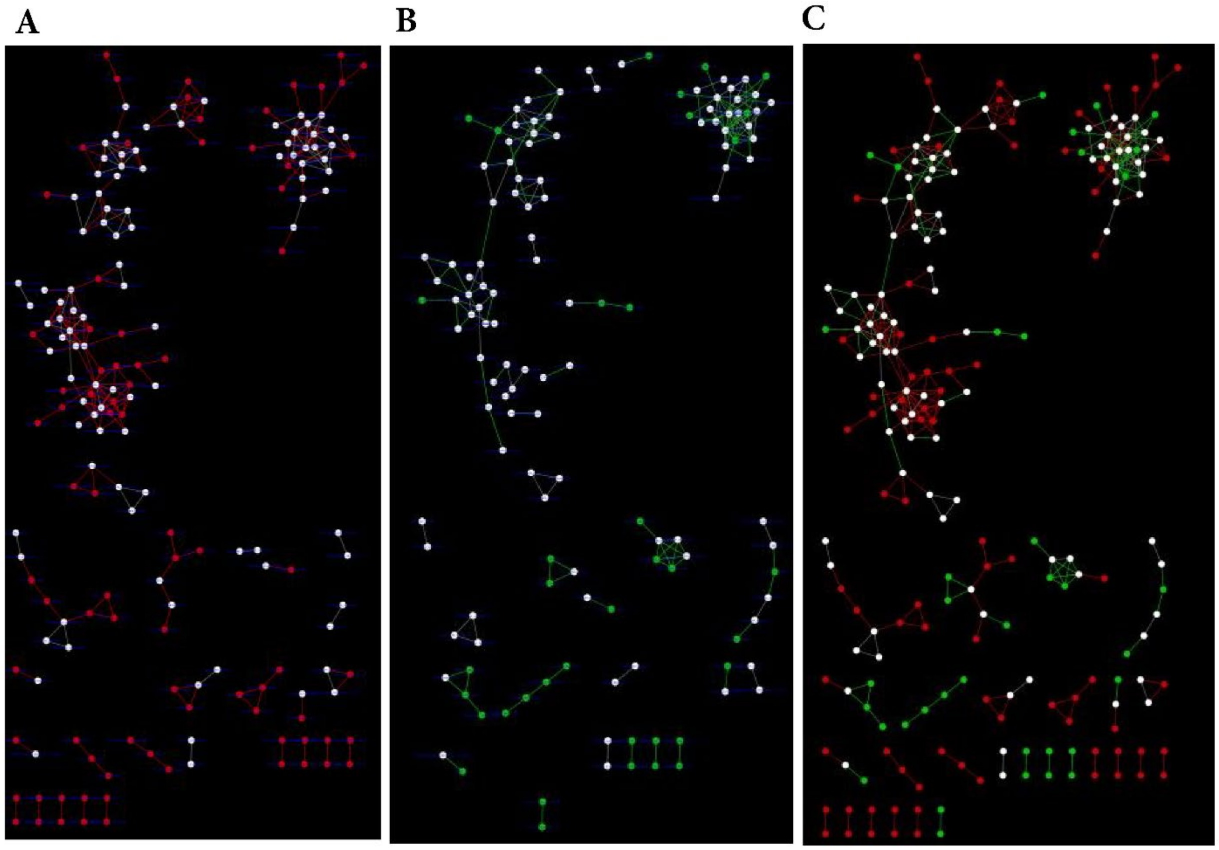

Supplementary Figure 8: DyNet visualization of co-occurrence networks of CRC and controls at the species level from the France dataset, (A) CRC network, (B) control network, and (C) synchronized CRC and control co-occurrence networks. Red nodes and red edges are present only in the CRC network, green nodes and green edges are present only in the control network, whereas white nodes are common in networks.

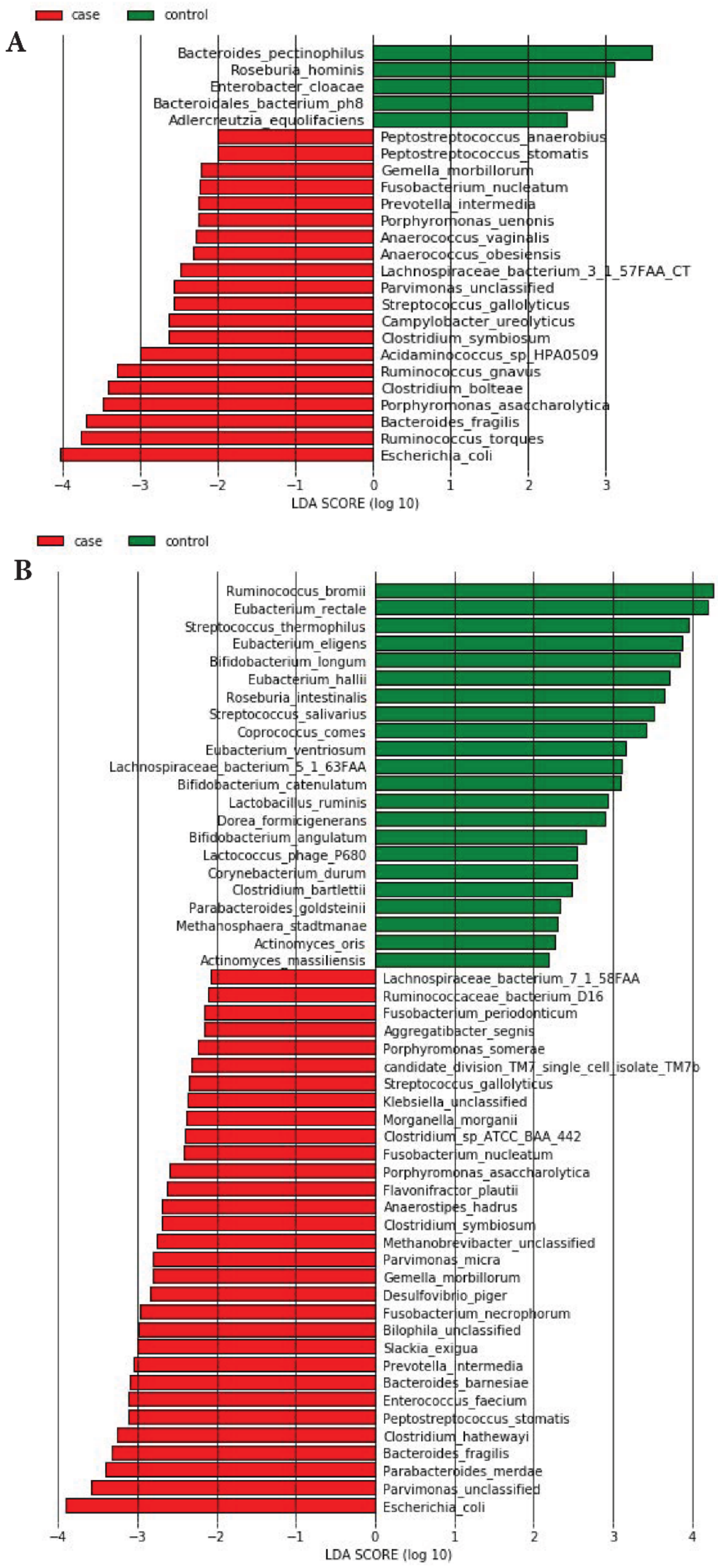

Supplementary Figure 9: Visualization of LEfSe identified differential abundant species between CRC and healthy controls. CRC enriched species are indicated with a negative LDA

score (red), and species enriched in healthy controls are indicated with a positive LDA score (green). Only species with the LDA score >2 at $p < 0.05$ are shown in the plot. (A) Histograms of differential species in USA dataset. (B) Histograms of differential species in France dataset.

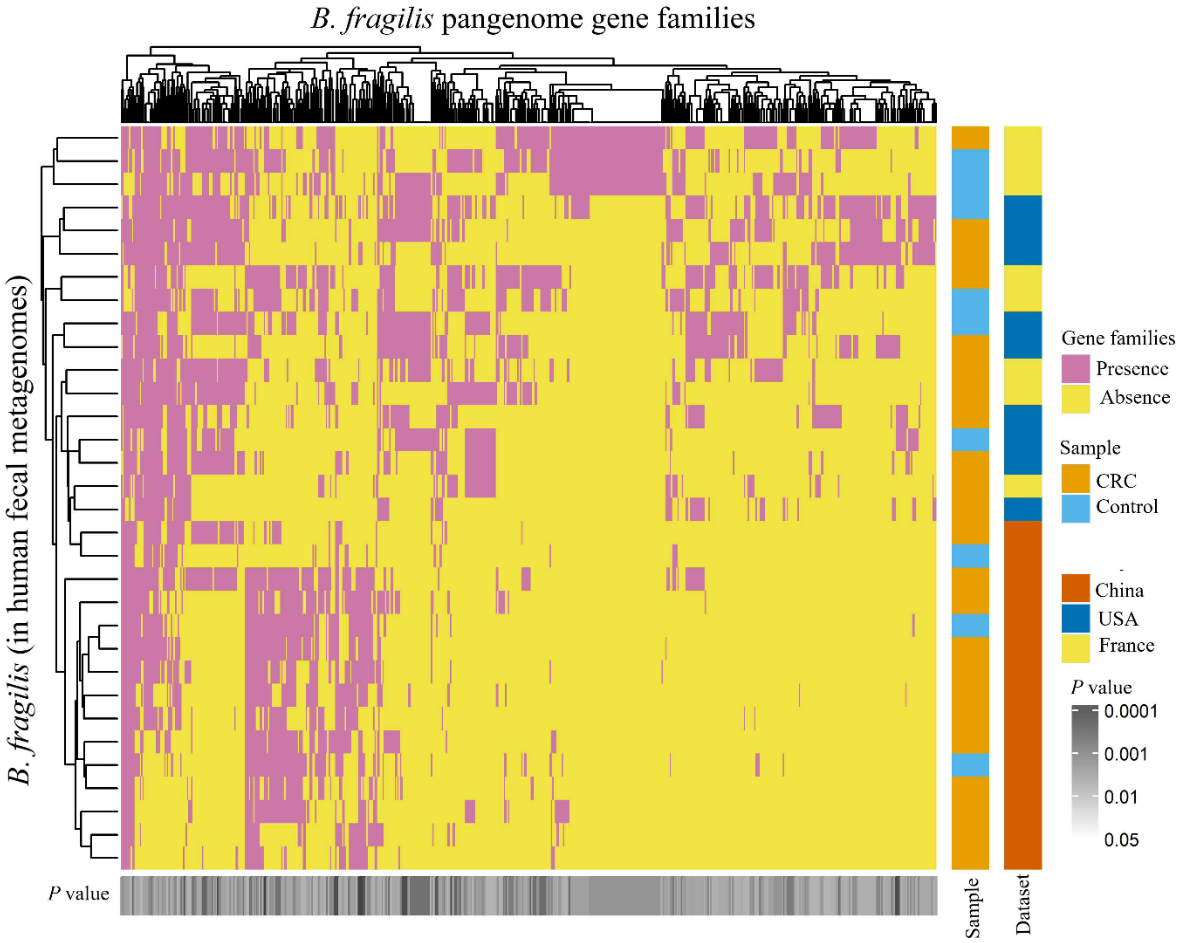

Supplementary Figure 10: Heat maps of the strain-level genomic diversity of *B. fragilis* across three geographic regions- USA, China, and France. The significant differential gene families (p-value < 0.05) were identified using Fisher exact test on presence and absence gene family profiles.

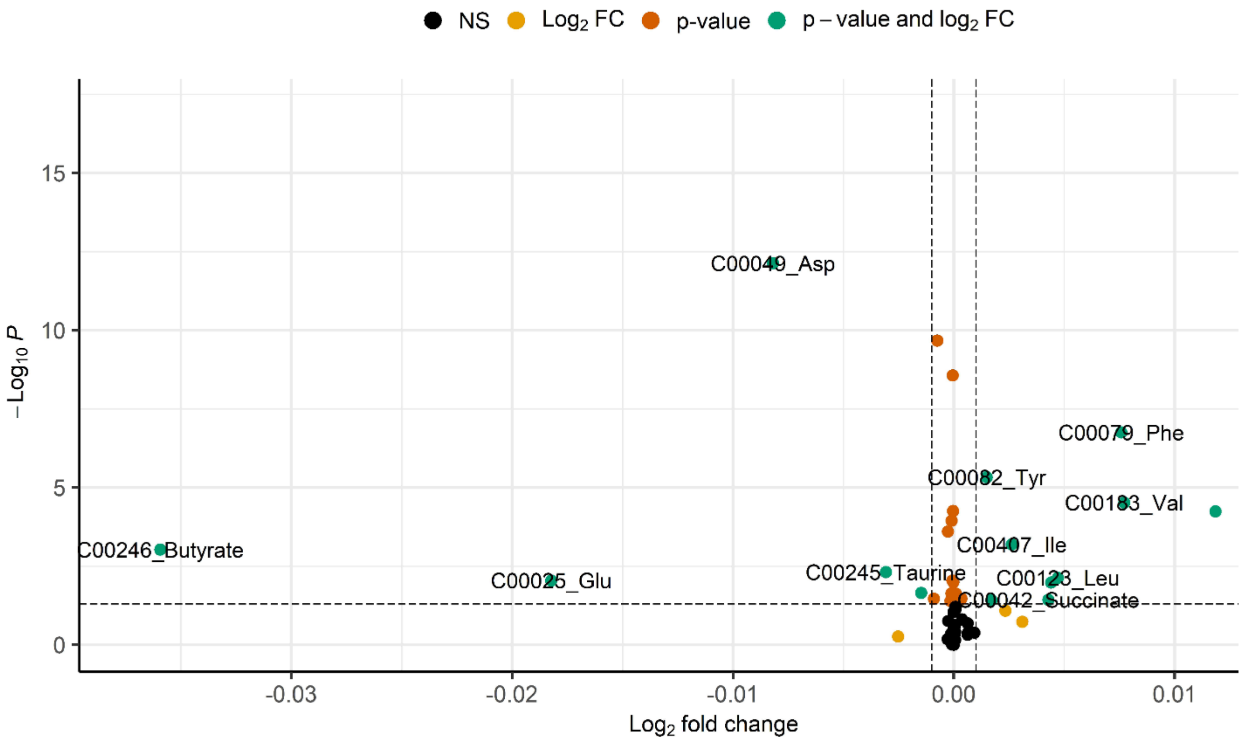

Supplementary Figure 11: Analysis of *MelonnPan* predicted metabolites in USA, China, and France datasets. Volcano plot showing differentially abundant metabolites between CRC and healthy control groups.
